# Genomic Diversity of *Streptomyces clavuligerus*: Implications for Clavulanic Acid Biosynthesis and Industrial Hyperproduction

**DOI:** 10.3390/ijms252010992

**Published:** 2024-10-12

**Authors:** Paula Ríos-Fernández, Carlos Caicedo-Montoya, Rigoberto Ríos-Estepa

**Affiliations:** 1 Grupo de Investigación en Simulación, Diseño, Control y Optimización de Procesos (SIDCOP), Departamento de Ingeniería Química, Universidad de Antioquia, Medellín 050010, Colombia; pnathalia.rios@udea.edu.co; 2 Grupo de Bioprocesos, Departamento de Ingeniería Química, Universidad de Antioquia, Medellín 050010, Colombia; candres.caicedo@udea.edu.co

**Keywords:** clavulanic acid biosynthesis, pan-genome analysis, *Streptomyces clavuligerus*, genomic profiling, metabolite hyperproduction

## Abstract

*Streptomyces clavuligerus* is a species used worldwide to industrially produce clavulanic acid (CA), a molecule that enhances antibiotic effectiveness against β-lactamase-producing bacterial strains. Despite its low inherent CA production, hyper-producing strains have been developed. However, genomic analyses specific to *S. clavuligerus* and CA biosynthesis are limited. Genomic variations that may influence CA yield were explored using *S. clavuligerus* strain genomes from diverse sources. Despite the slight differences obtained by similarity index calculation, pan-genome estimation revealed that only half of the genes identified were present in all strains. As expected, core genes were associated with primary metabolism, while the remaining genes were linked to secondary metabolism. Differences at the sequence level were more likely to be found in regions close to the tips of the linear chromosome. Wild-type strains preserved larger chromosomal and plasmid regions compared to industrial and/or hyper-producing strains; such a grouping pattern was also found through refined phylogenetic analyses. These results provide essential insights for the development of hyper-producing *S. clavuligerus* strains, attending to the critical demand for this antibiotic enhancer and contributing to future strategies for CA production optimization.

## 1. Introduction

The *Streptomyces* genus includes Gram-positive bacteria that have played a pivotal role in the discovery and production of secondary metabolites. These bioactive compounds encompass antibiotics and anticancer agents, as well as molecules with antifungal and immunosuppressive properties [1]. An exemplar is *Streptomyces clavuligerus*, cultivated in submerged cultures for the industrial production of clavulanic acid (CA). CA enhances the effectiveness of antibiotics by inhibiting β-lactamases, enzymes produced as a defense mechanism by bacterial strains resistant to β-lactam antibiotics [1]. Given the inhibitory property of CA and the exponential increase in β-lactamase-producing bacterial strains, CA is and will continue to be a highly demanded molecule [2].

While *S. clavuligerus* cells typically produce low quantities of CA, some hyper-producing strains have been developed through random mutagenesis and semi-rational design [3]. Comparative genomics and analysis of the pan-genome have served as significant tools in investigating *Streptomyces*, especially in the quest for new molecules with antibiotic properties and research on known molecules [4,5,6]. Despite decades of study, the molecular underpinnings governing CA production have not been fully understood, posing technical challenges to industrial-scale production, such as the need for stable high-producing mutants to maintain a consistent product quality and yield [7,8,9], the difficulty in achieving optimal fermentation conditions, including shear stress and oxygen transfer limitations [9], and managing the formation of by-products that inhibit CA production or complicate downstream processing [10]. 

Recent research in the context of CA production using *S. clavuligerus* includes applications of metabolic engineering, pathway-specific gene overexpression, the optimization of fermentation conditions, and systems biology approaches. For instance, overexpression of the glycerol utilization operon has significantly enhanced CA production [10]. Additionally, overexpressing regulatory genes like *ccaR* and *claR* has increased the CA yield by approximately 43% [11]. The optimization of fermentation conditions, such as fed-batch operations in bioreactors, has also improved production rates [9]. Integrating metabolic engineering with systems biology and implementing multi-omics approaches have been suggested to better understand and manipulate the metabolic and regulatory networks involved in CA production [1]. Nonetheless, the number of genomic analyses focused on *S. clavuligerus* and CA biosynthesis is limited [12], particularly in terms of understanding the strain-specific genomic differences in CA yields. The diversity of *S. clavuligerus* strains—each exhibiting variability in the amounts of CA produced—calls for a systematic and integrative approach. Our study addresses this gap by conducting an extensive pan-genome analysis of various *S. clavuligerus* strains, exploring the genomic factors influencing CA yield disparities.

There are 31 available *S. clavuligerus* strain genomes to date (August 2024), and this data set contains genomes of wild-type and industrial and/or overproducing strains, which have been shown to yield varying quantities of CA. These strains differ in their yields of CA, have been isolated from different regions (within China, Spain, USA, and UK), and their genomes have been published by laboratories from a variety of countries (Korea, The Netherlands, UK, and USA). These observations warrant a closer examination of the variability within the data set, aiming to elucidate potential findings relevant for CA production.

We performed a pan-genome analysis using the available *S. clavuligerus* genomes as inputs, focusing on characterizing the genome of this species and identifying key dissimilarities among strains at the genome level that might contribute to understanding the differences in the yields of CA produced. The results reported here may serve as guidance for the future development of novel strategies for developing hyper-producing *S. clavuligerus* strains.

## 2. Results

### 2.1. Characterization

We initially compared *S. clavuligerus* strains in terms of the CA produced based on data obtained from the literature (Supplementary Table S1). Of the downloaded genomes, 30 out of 31 genomes presented a completeness of ≥95% and were selected and used in subsequent analyses. The genome size and %GC content were obtained for the total scale, while the chromosome and plasmid scales were obtained when possible. The sizes of these 30 *S. clavuligerus* strain genomes ranged between 7.7 and 9.2 Mbp. Considering 16 out of 30 genomes (as 16 genomes were complete, depicting chromosome and plasmid sequences), the chromosome size ranged between 6.7 and 6.9 Mbp and the plasmid size varied between 10 kbp and 1.8 Mbp, while the total %GC content of the 30 genomes ranged between 71% and 72.5%, the chromosomal %GC content was 72.5% for all above-mentioned 16 genomes, and the %GC content of plasmids for these genomes ranged between 70% and 75%. Additional descriptive variables obtained from genome annotation per genome were the total CDS, rRNA, repeat regions, tRNA, and tmRNA. These results are summarized in Supplementary Figures S1–S3.

### 2.2. Pan-Genome Estimation

**Figure 1:**
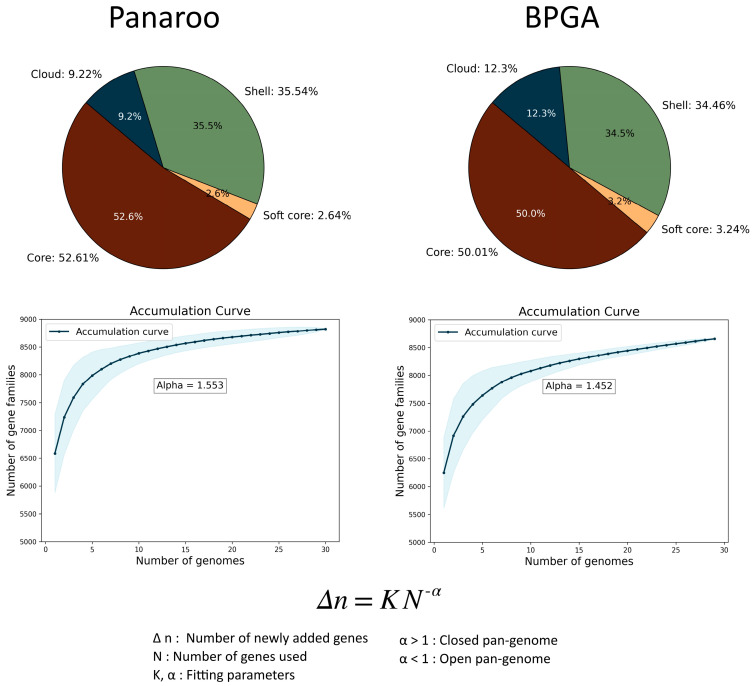
Pan-genome composition and rarefaction curves by Panaroo (**left**) and BPGA (**right**). According to Panaroo results, the pan-genome of *S. clavuligerus* contains 8821 clusters (4641 core genes, 233 soft-core genes, 3135 shell genes, and 813 cloud genes). The pan-genome was shown to be closed in both cases (α > 1) by performing 1000 permutations of genomes and fitting a power law to pan-genome counts.

According to the Panaroo results, the pan-genome of *S. clavuligerus* is closed (α > 1) with a size of 8821 clusters, of which 4641 are core genes, 233 are soft-core genes, 3135 are shell genes, and 812 are cloud genes. These results were similar to those obtained from the default setting iteration using BPGA. In such a case, the pan-genome is also closed (α > 1) with a size of 8716 clusters, with 4288 core genes, 283 soft-core genes, 3101 shell genes, and 1044 cloud genes (Figure 1). Using the Panaroo gene presence/absence matrix, we obtained a heatmap showing a pairwise comparison, applying hierarchical clustering with the Jaccard index (Supplementary Figure S4A) and Manhattan distance for comparison (Supplementary Figure S4B). ANI values were calculated for additional pairwise comparisons of all 30 genomes with FastANI, using the default parameters, for which the range of variation was between 99.37 and 100 (Supplementary Figure S4C). The previous analyses were also applied to the pan-genomes of *S. albidoflavus*, *S. olivaceus*, and *S. rimosus* for comparing the key parameters involved in the pan-genome estimation (number of genomes used, pan-genome openness, alpha value, and Jaccard/ANI index parameters), which are summarized in Table 1. Finally, we explored pan-genome visualization with PGGB, which performs all-to-all whole-genome alignment to identify variations, and then expanded its results using VG deconstruct for variant calling. For these steps, only complete genomes were used as inputs (16 out of 30 genomes); this subgroup of genomes included industrial, wild-type, and hyper-producing strains.

Among the multiple low-coverage regions (underrepresented regions in the graph due to their uncommonness across the genomes included) in the pan-genome graph results, three significantly large regions were highlighted. An inversion in four genomes detected through progressiveMauve alignment was also found by PGGB (Supplementary Figures S5 and S6); these genomes (GCF_015708605.1, GCF_015767815.1, GCF_015767895.1, and GCF_028752555.1) corresponded to wild-type and industrial strains (ATCC 27064, F1D14, F1D31, and DSM 738, respectively). Comparing strains based on path depth visualization showed that all industrial strain genomes had a low sequence depth at a certain chromosomal region (white region, blue rectangle in Supplementary Figure S6C). The hyper-producing strain genome (F613-1, GCF_001693675) also showed a low sequence depth at a certain plasmid region (green arrow in Supplementary Figure S6C). Having these two features was unique for this genome when compared to the rest of the genomes. For variant calling, we initially classified variants based on their size and obtained the two following groups: 3487 sequences shorter than 50 bp long and 9 sequences equal to or longer than 50 bp. The group of small variants was further classified according to type, as follows: Single-Nucleotide Polymorphism (SNP, 1166 variants), Insertion (909 variants), Deletion (63 variants), Multi-Nucleotide Polymorphism (MNP, 802 variants), and cases in which multiple variation types occurred (Others, 547 variants). Deletions occurred much more infrequently than any other type of variant; SNPs were more likely found in shorter sequences rather than in longer sequences, whereas variants classified as Others occurred often in sequences 10 bp long (Supplementary Figure S7). From the full set of variants, we identified 901 variants present within 892 chromosomal CDSs; the sequences of such variant-containing genes were annotated using KEGG. The annotation results of this set of genes (Supplementary Figure S8) suggest that differences at the nucleotide level among *S. clavuligerus* strains reside in proteins exclusively associated with the metabolism of carbohydrates, amino acids, proteins, lipids, and energy metabolism.

### 2.3. Phylogenetic Analysis

**Figure 2:**
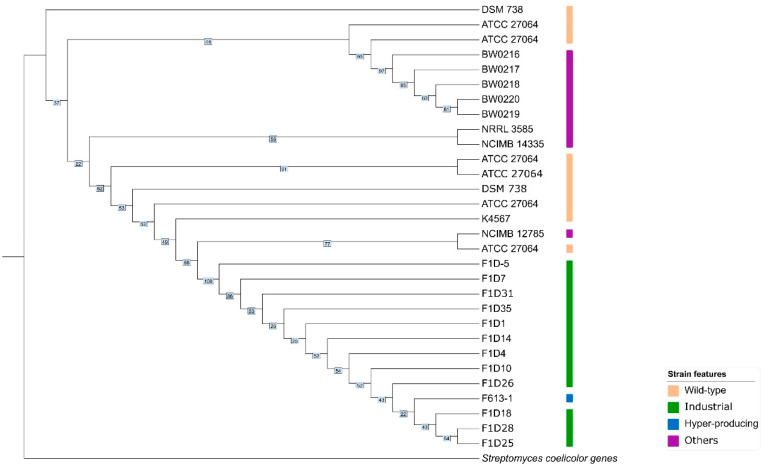
Evolutionary relationships of *S. clavuligerus* strains. Phylogenetic tree of concatenated sequences of 3760 core genes from *S. clavuligerus*. Wild-type, industrial, hyper-producing, and others strain types are depicted. Strain categories were defined as follows. Wild-type: strains reported by the literature to be wild-type. Industrial: strains known to be used in industrial CA production. Hyper-producing: industrial strains for which CA produced has been published and significantly exceeds that produced by a wild-type strain. Others: include strains known to have genetic modifications and/or whose relationship with wild-type strains is unclear and level of CA produced has not been published. Bootstrap values are used as branch support and highlighted in colored boxes. Additional information on CA produced per strain category can be found in Supplementary Table S1.

To investigate the evolutionary relationships between strains and genomes, we built three phylogenetic trees using different sequences obtained from the 30-genome dataset. The phylogenetic tree from an alignment of sequences annotated as 16S rRNA during genome annotation is shown in Supplementary Figure S9A; 16S rRNA sequences from *S. coelicolor* were used as an outgroup. Sequences annotated as 16S rRNA, *rpoB*, and *gyrB* were concatenated to form a three-sequence marker per genome; these markers were then aligned to build another tree, which is shown in Supplementary Figure S9B. A three-sequence maker using the same method was also built for *S. coelicolor* and used as an outgroup for this tree. Finally, we selected 3760 clusters classified as core genes in the previous pan-genome analyses, concatenated them to form a marker per genome, and aligned those sequences to build the tree shown in Figure 2; the outgroup is a marker built following the same steps but using *S. coelicolor* genomes. Overall, our approach involved a stepwise progression, as follows: initially, we individually analyzed commonly used genetic markers, then combined them into a single marker, and finally created a more comprehensive marker consisting of 3760 core genes. With this strategy, the complexity of the marker directly correlated with the clarity of discernible groups within the trees. The core gene tree shown in Figure 2 displays a group formed by industrial and hyper-producing strains, and another group formed by the remaining strains (‘wild-type’ and ‘others’); both groups displayed significant support values (100 and 95, respectively). Strains classified as ‘others’ or ‘wild-type’ did not form clearly defined groups. Thus, these were shown to be dissimilar among themselves and when compared to industrial strains. Despite this, ‘BW’ strains and a certain ‘ATCC 27064’ grouped with high support (branch support of 97 and 91, respectively). This cluster also included a hyper-producing strain, though support for its grouping with other industrial strains was low (branch support value of 22).

### 2.4. Functional Annotation and Streptomyces clavuligerus Gene Sets

We performed functional annotations of the core, shell, and cloud genes obtained in the pan-genome analysis using COG and KEEG databases. COG and KEGG annotation showed that, as the conservation of genes diminished (from core to cloud), so did the percentage of annotated sequences, as depicted in Table 2.

From KEGG annotation, the function of core and soft-core genes was mainly related to primary metabolisms such as those of carbohydrates, amino acids, and nucleotide metabolism, whereas shell and cloud genes were mostly associated with secondary metabolisms. A significant number of core genes belonged to the environmental information processing category, as expected for a species within the *Streptomyces* genera. Streptomycetes can utilize multiple environmental niches (sea, soil, and other organisms when living as symbionts) and are highly susceptible to environmental changes as, in response, they can display numerous morphological and molecular phenotypes, including their well-known capabilities for secondary metabolite production [13,14].

**Figure 3:**
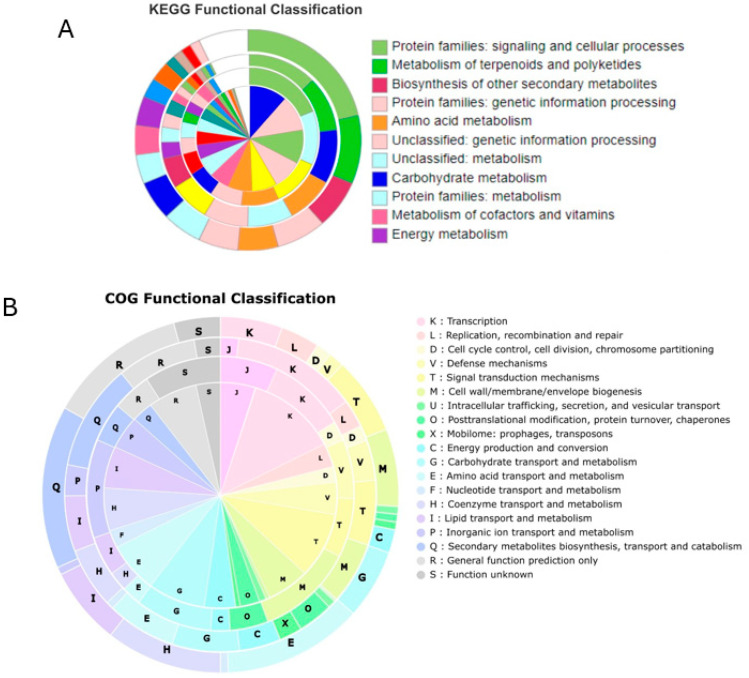
Functional annotation of *S. clavuligerus* pan-genome. Annotation of core (inner), soft-core, shell, and cloud genes (Outer) of *S. clavuligerus* pan-genome based on (**A**) KEGG Prokaryote database and (**B**) COG database.

The unclassified metabolism category was among the most represented categories for soft-core gene annotation. The most represented categories for shell and cloud genes included cellular signaling processes, the metabolism of terpenoids and polyketides, and the biosynthesis of secondary metabolites. Interestingly, environmental information processing was a part of all gene categories, except for cloud genes (Figure 3A).

From COG annotation, functional categories expected to be highly conserved, that is, those more likely to be represented by core and soft-core genes (such as replication, recombination, and repair (L), intracellular trafficking, secretion, and vesicular transport (U), and cell cycle control, cell division, and chromosome partitioning (D)), were represented in all gene types. Likewise, genes belonging to transcription category (K) appeared to be key for the species, as this category was among the most highly represented, regardless of gene category. Core and soft-core functional categories were the same; further, the variation among the different types of genes came from the number of genes assigned to each functional category. Such categories are mainly associated with primary metabolisms, while the shell and cloud categories included genes responsible for secondary metabolism processes. One of the highly represented functional categories for shell and cloud types was general function prediction only (R), indicating that the function of genes in such groups are yet to be determined. Coenzyme transport and metabolism (H) was one of the most frequent categories among these two gene types (Figure 3B).

**Figure 4:**
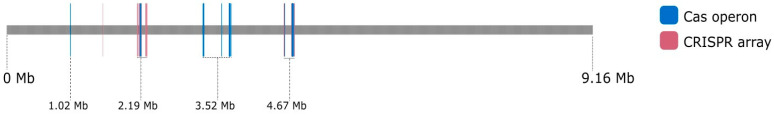
CRISPR-Cas systems identified in *S. clavuligerus* genomes. Complete genomes of *S. clavuligerus* strains were analyzed. The grey line represents positions within a hypothetical genome whose length is the maximum among genomes considered. Relative positions of Cas operons and CRISPR arrays identified in all genomes are represented as blue and red lines, respectively. Dotted lines indicate the average starting point of some subgroups.

CRISPR-Cas systems were identified in the *S. clavuligerus* complete genomes, where three CRISPR arrays, two Cas operons, and one CRISPR-Cas system per genome were found. The predicted type of system was I-B for all genomes. When comparing the results from all genomes, some of the CRISPR arrays and Cas operons were distant from each other, yet two groups of CRISPR arrays and Cas operons were found to be closed enough to form a system. Nonetheless, the essential criterion was the functional integration of the CRISPR array and Cas proteins for the adaptive immune response. In Figure 4, emphasis is placed on the relative starting positions of the two groups of CRISPR arrays and Cas operons, specifically those situated at the shortest distances from each other (2.19 Mb and 4.67 Mb).

**Figure 5:**
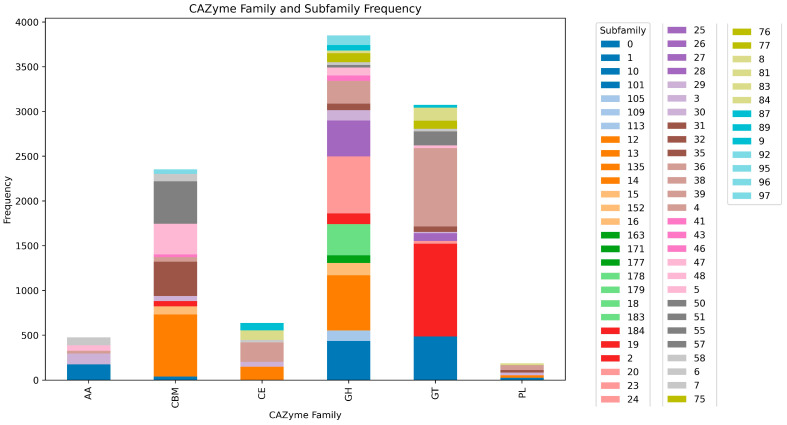
CAZyme families and subfamilies present in *S. clavuligerus* genomes. CAZyme families are displayed as stacked bars containing subfamilies (code numbers in legend). The counts per family are shown on y-axis.

The presence of CAZymes families and subfamilies in the full 30-genome set was assessed using dbCAN3. The most frequent family was Glycosyl Transferases (GT), while the family with the highest number of subfamilies was Glycoside Hydrolases (GH). The less frequent family was Polysaccharide Lyases (PL) (Figure 5).

We used 4629 core genes of *S. clavuligerus* as queries to search within a database of 145,452 sequences of *Streptomyces* core, soft-core, shell, and cloud genes obtained in a previous study [6]. From this search, 3522 *S. clavuligerus* core genes were identified, meaning that the remaining 1107 core genes can be considered as *S. clavuligerus* ‘fingerprints’. These fingerprint genes were annotated with the KEGG and COG databases as described above for other groups of genes (Supplementary Figure S10).

### 2.5. Biosynthetic Gene Clusters

**Figure 6:**
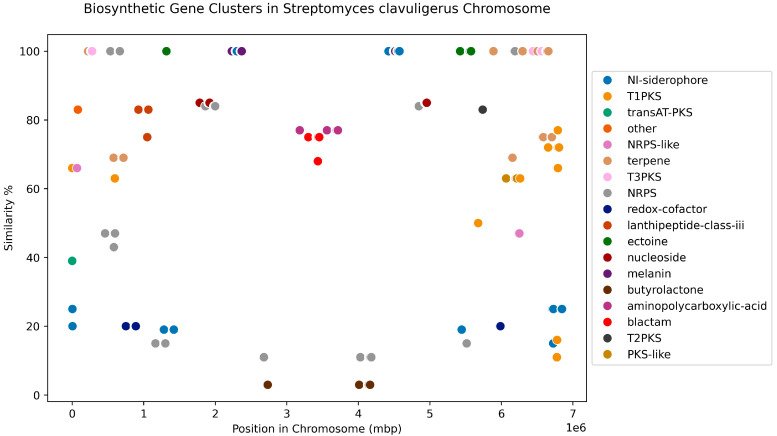
Type and relative position of BGCs found in the chromosome of *S. clavuligerus* genomes.

We ran antiSMASH on the full set of 30 genomes and 16 complete genomes to identify and compare the potential BGCs within them. In the full set of genomes, a total of 882 BGCs were found, and the average number of BGCs per genome was 28.3 (range of 36 to 20), with Terpenes and NRPS being the most represented BGC categories in the *S. clavuligerus* genomes. These results are summarized in a presence/absence matrix per genome of the gene function annotation that antiSMASH related to the BGC genes (Supplementary Figure S11); a total of 473 annotations were found. After comparing a wild-type strain complete genome to that of a hyper-producing strain, using such a gene function annotation matrix, 52 differences were found. We depicted the BGCs per chromosome and plasmids for the complete genomes. For this set, the average number of BGCs per chromosome was 22.7, while the average for plasmid sequences was 7.4. Figure 6 depicts the relative positions within the genomic sequences and similarities with known clusters per BGC within the chromosomes of these 16 complete genomes.

**Figure 7:**
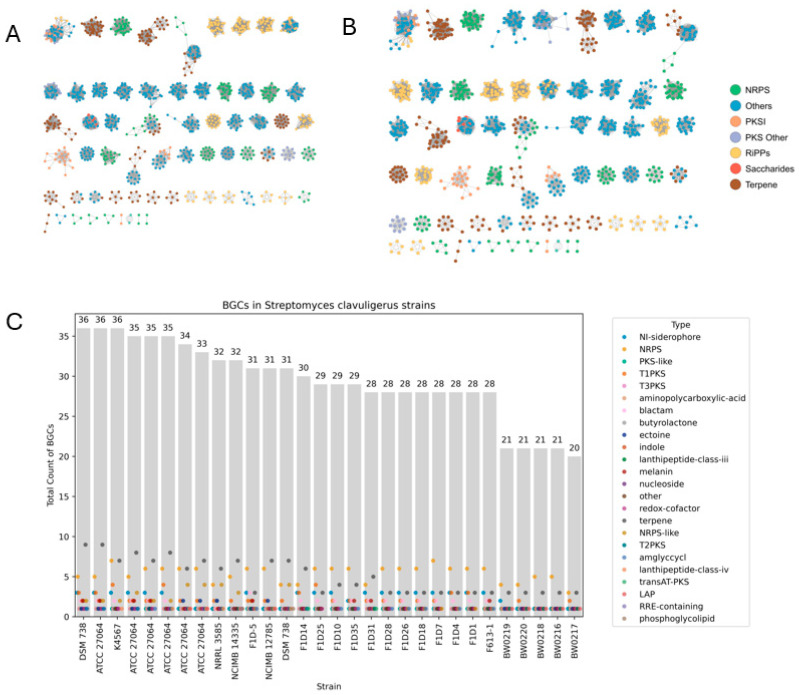
Biosynthetic gene clusters (BGCs) present in 30 *S. clavuligerus* genomes. BGCs annotated by BiG-SCAPE (**A**) and MIBiG (**B**), with each BGC type showcased in a different color. Annotation of BGCs by antiSMASH is also shown (**C**), with more specific categories.

We used BiG-SCAPE for the characterization of the BGCs in the 30 *S. clavuligerus* genomes and included annotations of the MIBiG database for comparison (Figure 7A,B). BGCs classified as Others appeared to be the most abundant and formed well-defined groups that were consistent in both sets of results. In contrast, Saccharides was the least-frequent BCG type in BiG-SCAPE (Figure 7A) and MIBiG (Figure 7B). Groups made of different classes of BGCs formed in both BiG-SCAPE and MIBiG; these groups contained BGCs classified as Others. When considering only those known BGCs, Terpene was the most abundant group in both iterations; this result was also found in the antiSMASH results for BGC comparisons among strains (Figure 7C). Those groups, including the lowest number of BGCs (found at the bottom of Figure 7A,B), showed significant diversity, as nearly all BGCs categories were represented. As for the antiSMASH results (Figure 7C), the following three groups were identified: one that included wild-type strains only (from 36 to 31 BGCs), except for F1D-5; a second group composed of industrial strains only, including a hyper-producing strain (from 30 to 28 BGCs); and a third group that included all ‘BW’ strains (between 21 and 20 BGCs). The former two groups included both complete and non-complete genomes, while the latter included only non-complete genomes.

### 2.6. Clavulanic Acid Cluster

**Figure 8:**
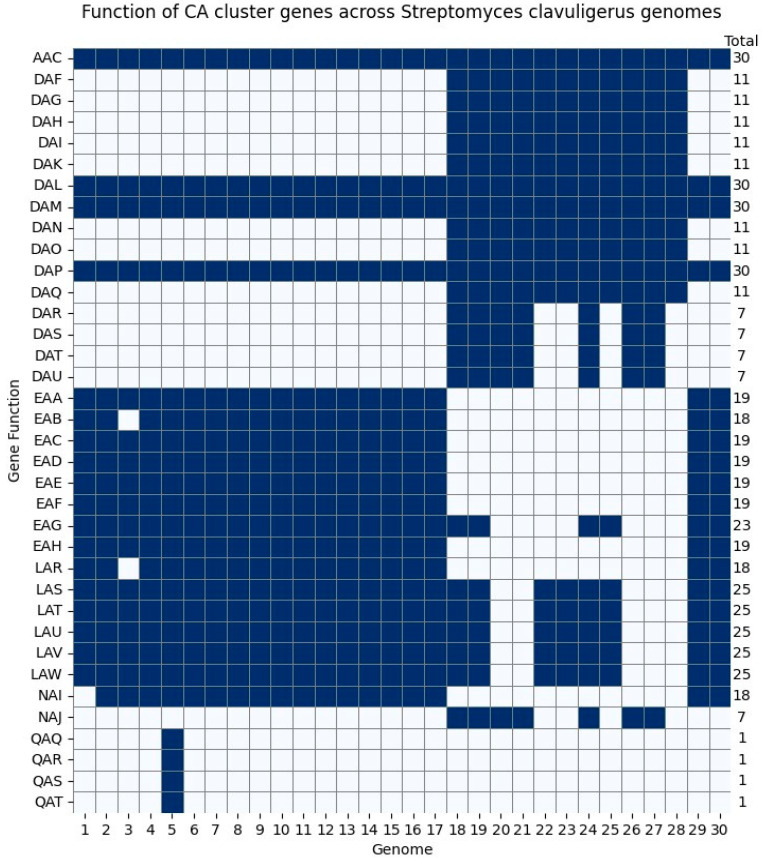
Presence/absence of genes within BGCs associated with CA biosynthesis of 30 *S. clavuligerus* genomes. AAC: Hypothetical protein, EA: Homoserine/homoserine lactone efflux protein, EAB: Flavin reductase, EAC: Aldo-keto reductase IolS, EAD: Clavaminate synthase 1, EAE: Homoserine O-acetyltransferase, EAF: 3,6-diketocamphane 1,6-monooxygenase, EAG: Putrescine--pyruvate aminotransferase, EAH: Serine/threonine-protein kinase PknD, LAR: Protein-glutamate methylesterase/protein-glutamine glutaminase, DAP: Glutamate N-acetyltransferase 2, LAS: Agmatinase, DAM: Carboxyethyl-arginine beta-lactam-synthase, DAL:N(2)-(2-carboxyethyl)arginine synthase, LAT: Fluorothreonine transaldolase, LAU:2-iminobutanoate/2-iminopropanoate deaminase, LAV: Methionine aminotransferase, LAW:L-threo-3-hydroxyaspartate ammonia-lyase, NAI:Vitamin B6 salvage pathway transcriptional repressor PtsJ, QAQ: Histidinol-phosphate aminotransferase, QAR: Putative N-acetyl-LL-diaminopimelate aminotransferase, QAS: Sugar efflux transporter, QAT:Adenosylmethionine-8-amino-7-oxononanoate aminotransferase, DAF: Transcriptional regulatory protein MoaR1, DAG: Beta-lactamase inhibitory protein, DAH:L-lysine-epsilon aminotransferase, DAI: Tyrocidine synthase 2, DAK: Peptidoglycan D,D-transpeptidase MrdA, DAN: Proclavaminate amidinohydrolase, DAO: Clavaminate synthase 2, DAQ: Heme-binding protein A, DAR: HTH-type transcriptional regulator ArgP, DAS: Putative oxidoreductase, DAT: Cytochrome P450-SU2, DAU:Ferredoxin-2, and NAJ: Putative amino-acid metabolite efflux pump.

The cluster associated with CA biosynthesis was analyzed from two perspectives, functional annotation and gene sequence. Figure 8 narrows down the antiSMASH presence/absence gene function annotation matrix (Supplementary Figure S11) to the level of gene functions within the BGCs associated with CA biosynthesis found in all genomes (regardless of whether they were in chromosome or plasmid sequences); it showed an industrial strain (genome 5 in figure) to have four different annotations within the cluster. Surprisingly, no differences between hyper-producing and wild-type strains were apparent; similar observations arose when examining the relative positioning of CA-related BCGs within the genomes of other CA-producing species, as detailed in Supplementary Table S2. The fact that only one of the industrial strains presented additional gene functions within the CA cluster compared to the rest and the lack of differences between the hyper-producing strain and the rest suggest that gene insertion in the CA cluster is not a common procedure for enhancing CA production in non-wild-type strains. Therefore, future experimental studies may explore genetic engineering strategies alternative to gene insertions at the CA cluster.

In terms of gene sequence analysis, antiSMASH identified 33 different gene/domain functions within the CA cluster (excluding genes annotated as ‘hypothetical protein’), of which 28 presented differences in at least one amino acid when comparing the sequences of CA clusters obtained from the 16 complete genomes. The group of 33 functions was composed of sequences classified as CDS (genes) or PFAM (protein domains) by antiSMASH. We describe below the groups that presented differences in their amino acid sequences and were not annotated as ‘hypothetical protein’, including both CDS and PFAM domain types.

The alignment of putrescine-pyruvate aminotransferase showed that some sequences had 19 additional amino acids compared to the rest of the sequences. Each genome appeared to have a pair of short and long versions of the same gene, except for two wild-type genomes (GCF_015912395 and GCF_015912435). The shorter sequence was in the chromosome, whereas the longer was in the plasmid. Interestingly, the same pattern was found in the carboxyethyl-arginine beta-lactam-synthase and the glutamate N-acetyltransferase 2 alignments; in these cases, some genomes had only one sequence in the chromosome. The genomes that presented duplication of the gene belonged to wild-type strains, and one industrial strain (F1D-5) in the case of carboxyethyl-arginine beta-lactam-synthase. The alignment of agmatinase sequences showed that all amino acids were conserved, except for a sequence found in a genome from the F1D-5 strain, which also included a set of amino acids not found in any other sequences; all sequences were found in the plasmid. Finally, the 2-iminobutanoate/2-iminopropanoate deaminase and the L-threo-3-hydroxyaspartate ammonia-lyase alignments showed one plasmid sequence per genome, yet the F1D-5 genome displayed duplication of these two genes within the plasmid.

In general, the gene sequence analysis for CDS groups showed a pattern of duplicating genes whose copies could be distinguished by length and source (chromosome and plasmid). The main difference found was that industrial strains lacked plasmid copies of certain genes (carboxyethyl-arginine beta-lactam-synthase and glutamate N-acetyltransferase 2). The lack of an extra copy of these genes may confer a disadvantage for CA production for industrial strains, as carboxyethyl-arginine beta-lactam-synthase is crucial in the early steps of CA biosynthesis, catalyzing the formation of deoxyamidinoproclavaminate from ATP and L-N2-(2-carboxyethyl)arginine. Without the plasmid copy, the overall enzyme activity might be reduced, potentially leading to a lower production rate of CA. Likewise, glutamate N-acetyl-transferase 2 is involved in the acetylation of glutamate, a precursor in the CA biosynthesis pathway. The absence of the plasmid copy could result in a decreased availability of N-acetylglutamate, thereby limiting the flux through the CA biosynthesis pathway and reducing CA yields. However, the higher production of CA in industrial strains, despite the lack of plasmid copies of these genes, might be explained by factors such as the enhancement of chromosomal copies of the genes, compensating for the absence of plasmid copies; the overexpression of key regulatory genes that exert their function on the chromosome copies; or the enhancement of precursor supply pathways. In future studies, comparing chromosome and plasmid copies of the above-mentioned genes through further sequence analysis or enzyme modeling could be useful for revealing additional differences in the CA cluster between wild-type and industrial strains.

The two-sequence pattern described above for CDS groups was also identified for some PFAM groups (GAF, AAA_16, Trans_reg_C, N(2)-(2-carboxyethyl)arginine synthase, AMP-binding_C, TPP_enzyme_N, TPP_enzyme_M, TPP_enzyme_C, GATase_7, and Arginase, ArgJ), as some sequences represented domains with additional amino acids that were also conserved among these longer sequences. At the same time, shorter sequences lacking these amino acids conserved this feature among them. A second type of alignment was found in the Aminotran_3, TauD, BTAD, AMP-binding, PP-binding, Condensation, and Asn_synthase alignments, where essentially three types of sequence per genome were identified, displaying the highest level of variability among all genes/domains studied. Other PFAM groups that did not show any of these features (Ribonuc_L-PSP, Aminotran_1_2, PALP, and AzlC,) did not present an apparent pattern. It is worth noting the differences among PFAM domains which are a part of genes in the CA cluster. TauD is a domain part of clavaminate synthase; it participates in oxidative reactions, specifically those that require 2-oxoglutarate (α-ketoglutarate) and iron(II) as cofactors. TauD alignment showed two copies present in the chromosomes of all genomes, except for some wild-type strains (ATCC 27064, BW016, BW017, BW018, NCIMB 14335, NCIMB 12785, and NRRL 3585). The condensation domain is found in β-lactam synthetase and catalyzes the formation of an amide bond in the β-lactam ring structure during the CA biosynthesis process. Interestingly, only wild-type strain genomes contained this domain, presenting three copies located in the chromosome. The arginase domain is part of proclavaminate amidinohydrolase; this domain catalyzes the hydrolysis of amidino groups from proclavaminate, a key intermediate in CA biosynthesis. All the industrial strains (except for F1D-5) presented one copy of this domain located in the plasmid, whereas most of the wild-type strains presented two, one in the chromosome and another in the plasmid. A summary of these results is available in Supplementary Table S3.

### 2.7. Synteny

The alignment of 16 complete genomes of *S. clavuligerus* indicated a massive inversion of a certain chromosome region in four strains and a significant level of variation in plasmid sequences. The alignment of chromosome sequences showed certain levels of variation at the tips of the chromosomes, while the central regions remained highly conserved among genomes and strains (Supplementary Figure S12). Moreover, the alignment of plasmid sequences showed greater differences compared to the results obtained from chromosome analyses, where inversions, gains, losses, and duplication events were more frequent (Supplementary Figure S13). We performed a third alignment that included all complete genomes of *S. clavuligerus* strains and complete genomes of *S. coelicolor* strains to investigate the conservation of regions at the central part of the chromosome. Despite being conserved, some regions at the center of the chromosome were shown to be mainly inverted (Supplementary Figure S14). By using antiSMASH, we detected certain regions related to β-lactam and/or clavulanic acid biosynthetic gene clusters in the genomes of known CA producers. The alignment of these key regions showed two groups, one within which longer regions were conserved (approximately 45 kb), and another one composed of shorter conserved regions (approximately 20 kb) (Supplementary Figure S15). Regions from other species showed a high similarity with those regions belonging to *S. clavuligerus* genomes (Supplementary Figure S16).

## 3. Discussion

### 3.1. Characterization of Streptomyces clavuligerus Genome

*S. clavuligerus* is a Gram-positive bacterium well known for its ability to produce CA; its metabolite titer varies depending on the strain type, growth conditions, and CA separation process. In this study, we aimed to identify key differences at the genomic level that could account for the variation observed in the CA produced by dissimilar *S. clavuligerus* strains. We compared 30 genomes of strains which, according to the literature, can produce different amounts of CA. Multiple approaches were used to assess the similarity in these genomes; pairwise ANI allowed for measurements of similarity among pairs of genomes, while pan-genome analysis included the alignment of all source genomes. Other comparison methods were applied to groups of specific genes that were shown to be related to CA production differences; such methods included phylogenetic analysis, functional annotation, biosynthetic gene cluster analysis, and synteny.

Seeking a general characterization of the *S. clavuligerus* genome, the mean size and %GC content values of the strain genomes used here are closer to those of *Streptomyces* species when compared to other Actinobacteria [15,16]. The range of variation in the genome size of 2 Mbp might correspond to the range of variation found at the plasmid level, given that the latter varied between 1.8 Mbp and 10 Kbp. This observation seems consistent with the %GC content results, where the variation in the total %GC content was attributed to the variation found at the plasmid level. Because *Streptomyces* have a high %GC content, it is more likely for codons to have a G or C at the third position, making TTA a rare codon in these species [15]. The transfer RNA responsible for interpreting the TTA codon in *Streptomyces* significantly influences phenotype traits such as morphology, development, and secondary metabolite production, particularly during the stationary growth phase [17]. A general aspect identified through synteny and the graphical representation of the pan-genome was the presence of a potential inversion in the chromosome of 4 out of the 16 complete genomes. A chromosomal inversion, with its presence in both wild-type and industrial strains, suggests that it is likely a naturally occurring genetic variation within the species. The occurrence of the inversion in wild-type strains indicates that it may provide certain adaptive advantages in natural environments, potentially affecting gene expression or other genomic functions that confer a survival benefit. Its presence in industrial strains further implies that this inversion might also play a role in traits relevant to their industrial application. Additional analyses, such as gene expression studies or functional assays, would be needed to determine the impact of the inversion on *S. clavuligerus*’ phenotype and fitness.

### 3.2. The Pan-Genome of Streptomyces clavuligerus Is Closed

Pan-genomes fall into the two following categories: open and closed. An open pan-genome tends to expand as new strains are sequenced, reflecting a high genetic diversity. In contrast, a closed pan-genome signifies a relatively stable gene repertoire that experiences minimal changes with the inclusion of new strains. The pan-genome of groups closely related to *S. clavuligerus* has been characterized as open (*Streptomyces* genera [4,5,6], *Streptomyces* species [18], and Actinobacteria species [19]). In contrast, the pan-genome reconstruction we obtained for *S. clavuligerus* is closed (α = 1.59); thus, new genomes would be less likely to introduce significantly different genes for this species. Nonetheless, an alpha value close to one is often considered to indicate a pan-genome that is still expanding as new genomes are added, but the rate of growth is relatively slow. Using a small number of strains can result in parameter estimates (α) with large standard errors, and these errors can lead to inconsistent conclusions about the degree of openness or closeness of a species [20]. In our study, comparing our findings, different *Streptomyces* species showed that the pan-genome openness (α) was markedly influenced by both the quantity of the genomes analyzed and the nucleotide identity parameters. There seemed to be a positive correlation between the number of genomes used in the pan-genome estimation and the alpha value, meaning that a pan-genome that uses a greater number of genomes is more likely to be classified as open (Table 1). Alternatively, the type of relationship between these two variables and nucleotide identity parameters (Jaccard/ANI minimum values) was not apparent (Table 1). In the case of *S. clavuligerus*, which exhibited a closed pan-genome, it can be inferred that the alpha value was influenced by the high degree of identity among the genomic sequences utilized in this study. Furthermore, the identification of core and accessory genomes of *S. clavuligerus* showed that approximately half of the genes were shared by all strains (core genes) (Figure 1); such equal proportions of core and accessory genes reflects the substantial genetic diversity and adaptive potential of the species. The presence of a significant number of accessory genes (4180 out of 8821) also suggests the potential for horizontal gene transfer (HGT) within this species [21]. This high-level interpretation of pan-genome estimation can be visualized in the pairwise comparison heatmap (Supplementary Figure S4A,B), where nearly half of the regions presented dissimilarities, while the other half remained consistent for all genomes.

We used the Jaccard index to assess the level of similarity between genomes in terms of gene presence/absence counts. This index is a ratio between the intersection of sets (the number of genes shared by a given pair of genomes) and the union of sets (the total number of genes of a given pair of genomes); an index closer to one indicates a high similarity, while an index closer to zero indicates a low similarity. The Jaccard index values of all possible pairs formed between the 30 genomes showed that the similarity ranged between 0.66 and 1.00, where values higher than 0.70 were more frequent, while those between 0.66 and 0.70 were less frequent (Supplementary Figure S4A). These results suggest that only certain pairs of *S. clavuligerus* strains represent most of the divergence within the data set, and that the impaired balance in the genomes available may have influenced the estimation of pan-genome openness. Supplementary Figure S4C shows the average nucleotide identity (ANI) values when comparing all 30 genomes; these results had a compact range of variation (from 99.37 to 100), which is expected for comparisons at the species level [20].

### 3.3. Patterns in the Evolution of Streptomyces clavuligerus Strains

The evolutionary relationships among the strains did not seem to be clear when using 16S rRNA sequences only, although a high branch support was obtained (Supplementary Figure S9A). Using concatenated markers helped to understand the relationships between strains; a high branch support was found at the base of such trees, as well as for certain clades. Groups formed by wild-type strains showed a high branch support (86 and 95), while those that included wild-type and industrial strains showed the opposite (38 and 28), indicating a distinction in the evolutionary history of these groups. Interestingly, a CA hyper-producing strain was found among the most basal wild-type genomes (Supplementary Figure S9B). A core genes tree provided insights into the relationship between the genomes and strains. In this case, the genomes of wild-type and industrial strains formed monophyletic groups, all with a high branch support. The CA hyper-producing strain mentioned above was found among the most basal wild-type genomes (Figure 2).

The linear chromosome of *Streptomyces* has been characterized to have conserved sequences at the center and variation or repeat regions at the ends of the chromosome [22]. The results from genome-wide alignment (Supplementary Figure S12) suggest that this is also the case for *S. clavuligerus*. However, when compared to another *Streptomyces* species, the conservation of sequences is less straightforward, as many of these small, conserved regions were inverted or translocated (Supplementary Figure S14). 

Given the importance of CA and the fact that *S. clavuligerus* is used in its production, we explored the differences at the sequence level among genes associated with its biosynthesis for the four following CA producers: *S. clavuligerus*, *S. viridis*, *S. jumonjinensis*, and *S. katsurahamanus*. A synteny comparing regions related to β-lactam and/or CA revealed two groups of sequences based on length (Supplementary Figures S15 and S16). Notably, some sections within regions presented a lower conservation among strains, regardless of the length of the region that they were part of; this observation holds true for sequences from all the species included. These differences at the sequence level may be relevant, given that the genes found within the clusters may play regulatory roles in biosynthesis (e.g., *claR* and *orf-21*) [3]. 

Four gene functions found only in 1 of the 30 genomes analyzed (Figure 8) belonged to an industrial strain (F1D-5 and GCF_003454755); such gene functions seem to be related directly or indirectly to the biosynthetic pathway of CA in *S. clavuligerus*. First, histidinol-phosphate aminotransferase (QAQ) catalyzes one step in the biosynthesis of histidine liberating 2-oxoglutarate, which is a precursor of the CA pathway and a cofactor of clavaminate synthase (*cas*), a key enzyme that participates in two steps of CA biosynthesis. N-acetyl-LL-diaminopimelate aminotransferase (QAR) participates in the biosynthesis of lysine, which can enter the urea cycle to produce L-arginine, another precursor in the CA biosynthetic pathway. Another gene function corresponds to a sugar efflux transporter (QAS), which facilitates the transport of sugars to the extracellular space; regulating the concentration of sugar within cells can promote the maximization of yield and the regulation of the osmotic stress in culture. Lastly, adenosylmethionine-8-amino-7-oxononanoate aminotransferase performs a step in the biosynthesis of biotin, which can regulate the activity of carboxylation enzymes in the TCA cycle whose effect in the CA pathway has been reported [23]. The results found when comparing the CA clusters at the sequence level revealed differences among the strains in terms of enzyme domains, which may have an effect in the synthesis of CA (Supplementary Table S3). According to the antiSMASH results, the total number of clusters/regions related to CA biosynthesis per genome varied between one and four. When a genome presented only one region, it was more likely to find this at one the tip of the chromosome. On the contrary, genomes that presented from two to three regions had one region located towards a tip of the chromosome and another located at its center (Supplementary Table S2). Given the conservation features mentioned above, those regions located at one tip of the chromosome were more likely to present variation, at least at the sequence level.

Comparisons at the sequence level of genes within the CA clusters aimed to identify more detailed differences that could not be found at the functional level (Figure 8). Some of the genes within the cluster are known to participate in the regulation of CA biosynthesis as transcription factors such as *claR* and *orf-21* [1,24]. There are genes outside of the cluster that are believed to play a pleiotropic role and regulate these factors, e.g., *ccaR*, *cas1*, *areB*, *scaR*, *adpA*, and *cagRS* [1]. A third group of genes involved in the regulation process play a less exclusive role, as these mainly control the primary metabolism resources, which are involved in other processes coupled with CA biosynthesis, like the stringent response; these genes may include *bldG*, *bldA*, *relA*, and *Spot* [25]. We aimed to identify and compare a full list of regulatory genes, including those inside and outside the CA cluster, yet reference sequences of the genes outside the cluster are scarce; thus, the search within genomes was not successful. Future studies aiming to identify these types of genes within *S. clavuligerus* genomes may utilize an extended source of query sequences. Nonetheless, we identified and compared the *claR* sequences among 16 complete genomes. The *claR* gene encodes a protein with a Trans_reg_C domain, which is essential for DNA binding and transcriptional regulation in CA biosynthesis. The *orf-21* gene is also part of the CA biosynthesis cluster, and it is believed to play a role in regulation as a putative sigma factor, yet its specific PFAM domains could not be found and its overall function has not been fully described [1]. Trans_reg_C domain alignment presented a duplication pattern among genomes, where a longer version of its sequence was found in the chromosome (74 amino acids) and a significantly shorter version (54 amino acids) was found in the plasmid. This pattern held true for all genomes and strains, except for FD1-5 (GCF_003454755), which presented an extra copy of the short sequence in the plasmid. Given that this domain participates directly in recognizing target sequences, its sequence is shorter, and it has a different amino acid composition in the plasmid, it might be that the chromosome and plasmid domains exert two different regulatory roles.

### 3.4. S. clavuligerus Primary Metabolism Genes Are Highly Conserved, While Those Related to Secondary Metabolism Are Less Conserved among Strains

It has been shown that Streptomycetes possess highly conserved metabolism-related genes, specifically those involved in the primary metabolism [4,5,6]. *S. clavuligerus* is no exception, as both methods of functional annotation showed that primary metabolism genes are a part of core and soft-core genes, while those related to the secondary metabolism are less conserved among strains, as these were classified as shell and cloud genes (Figure 3). Experimental results are consistent with this observation in the context of CA biosynthesis. When gene expression or environmental conditions are controlled, a common response of *S. clavuligerus* strains is to activate alternative metabolic pathways that compensate for genetic or environmental changes [24,26]. Likewise, a comparison of the BGCs present in the 30 *S. clavuligerus* genomes revealed a consistent pattern of diversity in the BGC types and contents among strains. The predominant BGC for this species (Others) stood out from the other established categories (Figure 7A,B), highlighting the remarkable potential of this species to be employed in the production of molecules beyond CA. In addition, Terpenes are relatively common BGCs in *S. clavuligerus* genomes, aligning with the prevalent trend at the genus level, as this type has been found among the most common ones [6,27]. When considering complete genomes only, the grouping pattern of strains per number of BGCs showed that industrial strains tended to have less BGCs compared to wild-type strains. Such a result could partially explain the differences between the CA produced among these two types of *S. clavuligerus* strains, as the availability of primary resources for metabolite production would be higher in those strains with less BGCs (Figure 7C). 

CAZymes, or Carbohydrate-Active enZymes, are essential enzymes involved in the synthesis, modification, and breakdown of complex carbohydrates. They encompass various enzyme families with specific functions, such as glycoside hydrolases and glycosyltransferases. CAZymes play critical roles in cellular processes like nutrient assimilation and energy metabolism [4]. Understanding their functions and evolutionary patterns is crucial for deciphering carbohydrate metabolism and exploring their biotechnological applications. While specific studies elucidating the repertoire and functions of CAZymes in *S. clavuligerus* are limited [4], the results we obtained (Figure 5) unveil a robust repertoire, particularly highlighting the diversity across families and subfamilies in the categories of Carbohydrate-Binding Modules (CBM), Glycoside Hydrolases (GH), and Glycosyltransferases (GT). These findings are particularly noteworthy, as the annotation of variant-containing genes reveals a predominant representation in carbohydrate metabolism (Supplementary Figure S8). The presence of multiple GH families (e.g., GH5, GH6, GH9, and GH16) suggests that *S. clavuligerus* can degrade plant cell wall components like cellulose and hemicellulose, enabling it to utilize plant-derived polysaccharides as carbon sources. Particularly, GH5 and GH6 are cellulases involved in breaking down cellulose into smaller glucose units. The GH9 family includes endoglucanases that degrade cellulose, particularly at internal β-1,4-glycosidic bonds. GH43 and GH48 are associated with hemicellulose degradation. These families can break down xylans (a major hemicellulose component) into xylose [28]. The function and abundance of these GH enzymes in *S. clavuligerus* genomes suggests their usage in sustainable waste management and resource recovery through the bioconversion of organic materials. The presence of other GH families suggests that *S. clavuligerus* can efficiently degrade starch and glycogen, allowing it to utilize these storage polysaccharides from plant and microbial sources, which include GH13, an α-amylase family involved in breaking down starch into maltose and glucose; the GH77 family, which includes enzymes like amylomaltase, which further processes maltose and related oligosaccharides; the GH15 family, which includes glucoamylases that degrade starch by cleaving glucose units from the non-reducing ends; and GH31 α-glucosidases that break down starch and other α-linked glucans [28]. Additionally, other GH families highlight *S. clavuligerus*’ ability to degrade a variety of complex polysaccharides, including those containing galacturonic acid, fucose, and mannose. Enzymes in GH28 (the polygalacturonase family) degrade pectin by hydrolyzing the galacturonan backbone. Other families that perform similar functions include GH29 fucosidases and GH35 β-galactosidases. In terms of carbohydrate synthesis and modification, the glycosyltransferases present in *S. clavuligerus* suggest that it can synthesize a variety of glycoconjugates (GT1, GT2, GT20, and GT28) essential for cellular communication, adhesion, and biofilm formation, potentially including those related to antibiotic production [28]. Although *S. clavuligerus* is primarily recognized for its production of CA, the presence of GT1 enzymes indicates that it may also perform the glycosylation of other secondary metabolites. For instance, GT1 enzymes might alter the structure of secondary metabolites produced by *S. clavuligerus*, resulting in the creation of new glycosylated compounds with antibacterial or antifungal activities. The specific modification of carbohydrates is another potential capability of *S. clavuligerus*, and CE3 and CE4 enzymes are involved in deacetylating polysaccharides, which can modulate their solubility and degradation, while CE7 and CE9 enzymes may target specific ester-linked groups on carbohydrates [28]. These observations underscore the significant role of carbohydrate-related processes within the genetic makeup of *S. clavuligerus*.

CRISPR–Cas systems are classified by Cas protein differences and effector module sequence variations. Despite minimal sequence conservation, types I, III, and IV share similar class 1 effector complex organizations for pre-crRNA processing and target recognition. The distribution of the six types of CRISPR-Cas systems in the major archaeal and bacterial phyla showed that type I was the most common within Actinobacteria [29]. Given the capabilities of Streptomycetes for bioactive molecules, protocols that rely on CRISPR-Cas systems have shown promising results in gene expression control, genetic engineering, and gene screening in species of this genus [30,31,32]. The prediction of CRISPR-Cas systems in *S. clavuligerus* genomes revealed only one system of the I-B type; although this number is consistent with results for other species, the class of system differs [33,34]. I-B is considered to be a minimal subtype, because it lacks the essential interference component Cas3, suggesting functions beyond adaptive immunity. It is believed that these minimal type I variants may support guide-RNA-dependent transposition, a notion recently substantiated by experimental findings [30]. Additionally, an interference-deficient subtype I-E CRISPR–Cas system variant occurs in a different *Streptomyces* bacterium, not associated with mobile genetic elements, but linked to a gene encoding a STAND superfamily NTPase, which implies potential involvement in signal transduction, dormancy induction, or programmed cell death [34].

In a recent study [22], proteins exclusively found in each species (fingerprint) were identified for numerous Streptomycetes, including *S. clavuligerus*. In such study, an approximate number of 200 proteins were found to be exclusive to *S. clavuligerus*, while another study [4] found 114 strain-specific proteins for the F613-1 strain. Here, we identified 1107 potential fingerprint genes for this species. KEGG annotation revealed that most of these genes are linked to primary and secondary metabolisms. Yet, the most common categories were genetic information processing and signaling and cellular processes (Supplementary Figure S10A); COG annotation was consistent in demonstrating the association of these genes mainly with the primary metabolism. In this case, the most represented categories were transcription and amino acid transport and metabolism (Supplementary Figure S10B). Regardless of the database used, the categories of carbohydrate metabolism, nucleotide metabolism, and the category that included genes of an unknown function were identified (Supplementary Figure S10).

### 3.5. Future Perspectives

The pan-genome analysis indicated a remarkable variability within the genomes of *S. clavuligerus* strains, as nearly 50% of the genes were not present in all the strains/genomes studied. While the CA cluster was conserved across the *S. clavuligerus* strains in terms of functions, significant variability existed in terms of gene sequence and structural organization. For instance, industrial strains tended to lack plasmid copies of key biosynthetic genes such as carboxyethyl-arginine beta-lactam synthase and glutamate N-acetyltransferase 2, both of which play pivotal roles in the early steps of CA biosynthesis. In contrast, many wild-type strains exhibited a duplication of these genes, with one copy located in the chromosome and another in the plasmid. This duplication potentially increases the availability of key precursors and intermediates in the CA pathway, offering a mechanistic explanation for the higher CA yields in some wild-type strains. Despite this, industrial strains still produce high levels of CA, suggesting that compensatory mechanisms—such as enhanced expressions of chromosomal gene copies or regulatory overexpression—may mitigate the absence of plasmid-encoded genes.

Additionally, a sequence analysis of the proteins involved in CA biosynthesis uncovered subtle but potentially impactful variations at the amino acid level. For example, certain enzymes, such as putrescine-pyruvate aminotransferase, carboxyethyl-arginine beta-lactam-synthase, and glutamate N-acetyltransferase 2, exhibited additional amino acids in specific strains, which may affect enzyme stability, catalytic efficiency, and regulation. This variability highlights potential targets for metabolic engineering, where optimizing or modifying these enzymes could lead to an enhanced biosynthetic efficiency and increased CA production. The discovery of conserved domains within the CA cluster —such as those involved in β-lactam ring formation (e.g., condensation domains of β-lactam synthetase) and precursor supply pathways—suggests candidates for targeted genetic manipulation. The structural differences in the plasmid sequences between strains, along with the discovery of massive chromosomal inversions and regions of a low sequence depth in industrial strains, provide additional clues about genome architecture’s role in CA production. These findings suggest that future work could explore chromosomal rearrangements or plasmid engineering as strategies for stabilizing high-yielding strains (e.g., evaluating the effect of the removal of complete or partial pSCL4 plasmids, as depicted in the synteny analyses).

## 4. Materials and Methods

### 4.1. Input Data

We used all genomes available in NCBI [35] for *S. clavuligerus* as inputs (31 to date). The completeness of the genomic data was evaluated with BUSCO v5.4.5 [36] using a cut-off value of >95%. Genomes that surpassed the BUSCO threshold were annotated with Prokka v1.14.5 [37] to avoid annotation bias. Raw genome data were used to estimate the total size, total GC content, and size and GC content per genomic item (chromosome and plasmid). CDS, rRNA, repeat regions, tRNA, and tmRNA counts were obtained based on the annotation results. Metadata related to each genome and strain were downloaded from NCBI and collected from the public literature.

### 4.2. Pan-Genome Estimation

The pan-genome of *S. clavuligerus* was estimated with Panaroo v1.4.2 [38] and BPGA v1.3.0 [39], using data from 30 genomes as inputs. Panaroo employs a graph-based approach to represent the pan-genome, which can lead to more accurate reconstructions compared to methods based on sequence alignments or clustering alone; Panaroo also uses standard input (gff3) and output formats (gml, fasta, and csv); thus, it is easy to incorporate it into different workflows. No significant differences were observed upon modifying the parameters in Panaroo (mode, matching, re-find, gene alignment, and graph correction). Therefore, the results obtained from the default parameter settings of Panaroo were used for subsequent analyses, and BPGA results were used for comparison. Additional pan-genome estimations were performed for other *Streptomyces* species (*S. albidoflavus*, *S. olivaceus*, and *S. rimosus*) for comparison with the *S. clavuligerus* results of the pan-genome openness estimation. The complete genomes (16 out of 30) were used as inputs for PGGB v0.5.4 [40] to generate a pan-genome graph representation under default parameters. The gfa output file (Graphical Fragment Assembly) and the chromosome sequence of a wild-type strain (GCF_015708605) were used as inputs (pan-genome graph, reference contig) for variant calling analysis with VG v1.53.0 [41], which rendered variant types (single-nucleotide polymorphism, insertion, deletion, and multi-nucleotide polymorphism) across all complete genomes using the GCF_015708605 chromosome as a reference. The output file of the variant calling analysis (vcf) and the annotation files of such genomes (gff and faa) were employed for mapping the positions of variants within chromosomal CDSs and obtaining the sequences of the genes that contained them for further analysis.

### 4.3. Phylogenetic Analysis

We used three sets of sequences for phylogenetic analysis as follows: 16s rRNA sequences, concatenated 16S rRNA, *rpoB*, and *gyrB* sequences, and concatenated core genes (3760). Each set of sequences was aligned with MAFFT v7.520 [42] using the default parameters, while the -add function was used to integrate the outgroup in each case. For all trees, IQ-TREE v1.6.12 [43] was used for phylogeny inference with maximum likelihood; ModelFinder (module in IQ-TREE v1.6.12) [44] identified the best free rate variation models, and branch support was calculated using Ultrafast Bootstrap approximation [45].

### 4.4. Functional Annotation and Streptomyces clavuligerus Fingerprint

We annotated the core, soft-core, shell, and cloud gene sets obtained from the pan-genome analysis. The input sequences for functional annotation were obtained from the fasta reference file returned by Panaroo. Since paralogous gene clusters are represented only once in such files, the number of total clusters decreased (core: 4629, soft-core: 232, shell: 3012, and cloud: 729) when compared to the initial pan-genome results depicted in the pan-genome estimation section. The amino acid sequences per gene category were used when needed and were obtained by selecting the longest sequence between the translated nucleotide sequences in all six reading frames.

We used the Prokaryote KEGG database for annotation of the gene sets, where sequence-to-database mapping was performed by the BlastKOALA server v3.0 [46]. COGclassifier v1.0.5 python package [47] was used for COG annotation. We used CRISPRCasTyper v1.0.13 [48] to identify potential CRISPR-Cas systems in the *S. clavuligerus* complete genomes using the default search parameters. The identification of carbohydrate active enzymes (CAZymes) was achieved with dbCAN3 v4.1.4 [49], where the faa and gff files obtained from Prokka annotation were used as inputs and the CGC-Finder algorithm enabled a CAZyme gene clusters search. The result files from all 30 genomes were employed to depict the CAZymes family and subfamily frequencies using in-house scripts.

Genes encoding proteins unique to a given species are considered to be ‘fingerprints’ [22]. With the aim of searching for such genes in *S. clavuligerus* we, (i) created a local BLAST [50] database using core genes of *Streptomyces* genera obtained in a separate study [6], and used it to (ii) perform iterative search runs with each of the *S. clavuligerus* core gene sequences as queries using the Blastn [51] algorithm. Those core *S. clavuligerus* genes not found in the local database (core genes of *Streptomyces* genera) were considered to be potential ‘fingerprint’ genes.

### 4.5. Biosynthetic Gene Clusters

antiSMASH v7.1.0 [52] was used for predictions of BGCs’ presence in the 30 genomes using a relaxed detection strictness and default parameters, enabling a Cluster Pfam analysis and Pfam-based GO term annotation. We obtained 1173 genebank files which were processed in BiG-SCAPE v1.1.2 [53] for a similarity comparison using default parameters and including annotations based on the MIBiG database. From these results, network files were used for visualization in Cytoscape [54], where nodes corresponded to BGCs and edges represented the level of similarity among them.

### 4.6. Clavulanic Acid Cluster

The following methods describe the steps taken for identifying potential differences at the sequence level of genes within the CA clusters of the 16 complete genomes. The input CA cluster amino acid sequences were obtained from the antiSMASH gene annotation results, which depicted the potential function of each gene/domain within the clusters. We grouped sequences based on gene/domain function and aligned them using MAFFT -auto mode. Those sequences whose function was ‘hypothetical protein’ were placed into a single group, while the rest of sequences were grouped according to their corresponding functional annotation. Sequences within a given group were aligned, and if a difference of at least one amino acid was found, the group was classified as differential. The group of ‘hypothetical protein’ sequences was treated differently, as an iterative two-step approach was applied to obtain more specific groups, as follows: (i) all-to-all alignments were performed and (ii) the groups (pairs in the first iteration) of sequences whose sequence identity was equal to or greater than 80% were selected (a criteria commonly used for amino acid sequences). These two steps were repeated until no groups could be formed due to not fulfilling the identity threshold. Those sequences that could not be part of a group of at least 20 sequences were placed into a separate group called ‘ungrouped hypothetical proteins’ and classified as differential. Each group that contained at least 20 sequences was treated as described for the non-hypothetical protein groups.

### 4.7. Synteny

We performed five different genome alignments using progressiveMauve v2.4.0 [55]. For the first alignment, only complete genomes of *S. clavuligerus* strains were used (16 out of 30). Then, we obtained two additional alignments, one that included chromosome sequences only, and another one that included plasmid sequences only. We performed a third alignment that included all complete genomes of *S. clavuligerus* strains and complete genomes of *S. coelicolor* strains. Finally, a synteny of key genomic regions associated with β-lactam and/or clavulanic acid gene clusters was obtained. Such regions were gathered from the antiSMASH results for the 30-genome set previously obtained, along with results from the same analyses performed for other CA-producing bacteria, such as *Saccharomonospora viridis*, *Streptomyces jumonjinensis*, and *Streptomyces katsurahamanus*. A total of 78 regions were included, where 75 corresponded to *S. clavuligerus* genomes and 3 corresponded to each of the other species’ genomes. 

**Figure 9:**
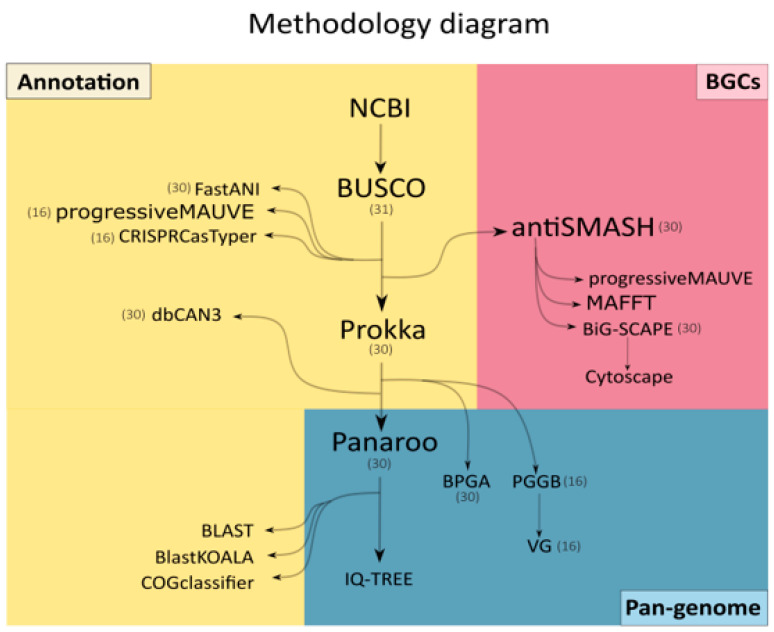
Diagram of the methodology displaying the tools, inputs, and output flow per step. The results can be categorized in Annotation analyses, Biosynthetic Gene Cluster analyses (BGCs), and Pan-genome analyses. The numbers correspond to the number of genomes used as input data for a given tool; no number is displayed in steps where input data were not genomes.

A summary of the methodology is shown in Figure 9.

## 5. Conclusions

- The genomes of *S. clavuligerus* strains exhibit varying degrees of similarity, with Jaccard index values ranging from 0.66 to 1.00, indicating that certain pairs of strains represent most of the divergence within the dataset. Differences in evolutionary history are observed between wild-type and industrial strains, with the latter showing less evolutionary clarity. The variability in genome size and %GC content is likely attributed to differences at the plasmid level, affecting strain-specific phenotypic traits such as CA production.
- The characterization of the pan-genome of *S. clavuligerus* as closed raises questions about the stability of its gene repertoire, especially considering the high genetic similarity observed among the 30 genomes analyzed. Approximately half of the genes are core genes shared across all strains, yet the addition of new strains would, indeed, bring about minimal changes in the gene pool. Moreover, the notable presence of accessory genes hints at the potential for horizontal gene transfer, which may contribute to the overall genetic diversity within *S. clavuligerus*.
- The diverse repertoire of CAZymes, including various glycoside hydrolases and glycosyltransferases, underscores *S. clavuligerus’*s capability to degrade and utilize a wide range of complex carbohydrates. This enzymatic versatility highlights its potential in sustainable waste management and resource recovery and suggests significant biotechnological applications, particularly in the synthesis of novel glycosylated compounds with antimicrobial properties.
- The presence of a minimal I-B type CRISPR-Cas system in *S. clavuligerus* indicates functions beyond traditional adaptive immunity, potentially involving guide-RNA-dependent transposition. This, coupled with the occurrence of an interference-deficient subtype I-E CRISPR-Cas system in related species, suggests a broader functional spectrum of CRISPR-Cas systems in Streptomycetes, encompassing gene regulation and signal transduction.
- *S. clavuligerus* exhibits a high diversity in BGCs, with primary metabolism genes being highly conserved and secondary metabolism genes showing more variation. This diversity in BGCs reflects the species’ potential for producing a wide range of molecules beyond CA. Differences in CA biosynthesis among the strains are linked to variations in gene clusters, particularly at the sequence level, which may influence regulatory mechanisms and enzyme function, impacting CA production.
- CA production by *S. clavuligerus* may be influenced by differences in gene clusters, particularly those related to regulatory elements and enzyme domains within and outside the CA biosynthetic cluster. These genetic variations could impact the efficiency of CA biosynthesis across different strains, highlighting the critical role of both chromosomal and plasmid-borne genes in optimizing CA yield.

## Figures and Tables

**Table 1:** Openness of four *Streptomyces* pan-genomes.

Species	N° of Genomes	Openness	Alpha	Minimum Jaccard Index Value	Minimum ANI Value
*S. clavuligerus*	30	closed	1.59	0.66	99.33
*S. rimosus*	57	closed	1.03	0.59	96.4
*S. albidoflavus*	64	open	0.83	0.63	95.73
*S. olivaceus*	70	open	0.86	0.74	97.75

Rows are pondered from lowest to highest number of genomes used for pan-genome estimation. Completeness of all genomes was estimated with Busco, Prokka was used for annotation, and pan-genome estimations were performed using Panaroo.

**Table 2:** Level of gene annotation per gene category and database.

		Annotation Level			
		(Percentage|Number of Annotated Genes)			
Gene Category	Total Number of Genes in Category	COG		KEGG	
Core	4629	72.30%	3349	40.50%	1873
Soft-core	232	65.50%	152	33.60%	78
Shell	3012	48%	1447	17.40%	524
Cloud	729	32.90%	240	9.60%	70

Percentage and proportion values represent the number of genes annotated out of the total number of genes that belong to a given category.

## Data Availability

The datasets employed for this study can be found in the Reference Sequence (RefSeq) database (https://ftp.ncbi.nlm.nih.gov/genomes/refseq/bacteria/ (accessed on 7 October 2024)). The accession number for the genomes included can be found in the Supplementary Table S3.

## Supplementary Materials

The following supporting information can be downloaded at: https://www.mdpi.com/article/10.3390/ijms252010992/s1.
