# Supplementary material for: Genomic Diversity of Streptomyces clavuligerus: Implications for Clavulanic Acid Biosynthesis and Industrial Hyperproduction
Source: Int J Mol Sci. 2024 Oct 12;25(20):10992. doi: 10.3390/ijms252010992 (PMC11507055; doi:10.3390/ijms252010992)
Supplement: Supplementary file 1 [file ijms-25-10992-s001.zip › Supplementary_Tables_Figures_ijms-3235348_Proof.pdf]

Supplementary material

Genomic Diversity of *Streptomyces clavuligerus*: Implications for Clavulanic Acid Biosynthesis and Industrial Hyperproduction

Paula Ríos-Fernández [1], Carlos Caicedo-Montoya [2] and Rigoberto Ríos-Estepa [1,*]

1 Grupo de Investigación en Simulación, Diseño, Control y Optimización de Procesos (SIDCOP), Departamento de Ingeniería Química, Universidad de Antioquia, Medellín 050010, Colombia; pnathalia.rios@udea.edu.co
2 Grupo de Bioprocesos, Departamento de Ingeniería Química, Universidad de Antioquia, Medellín 050010, Colombia; candres.caicedo@udea.edu.co
* Correspondence: rigoberto.rios@udea.edu.co

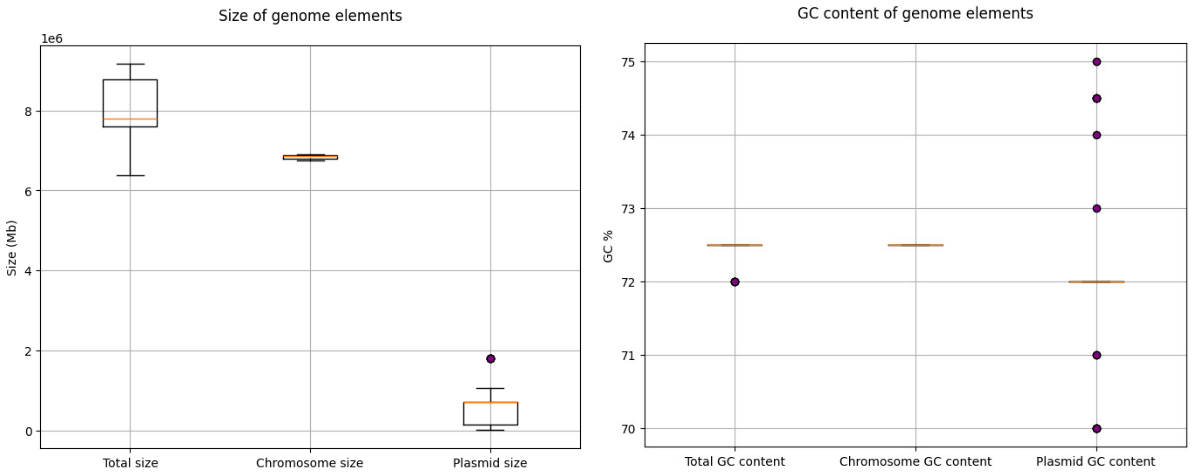

Figure S1. Genome, chromosome, and plasmid size and GC% of genomes. All 31 genomes are considered for total size and GC content, while 18 genomes account for chromosome and plasmid size and GC content.

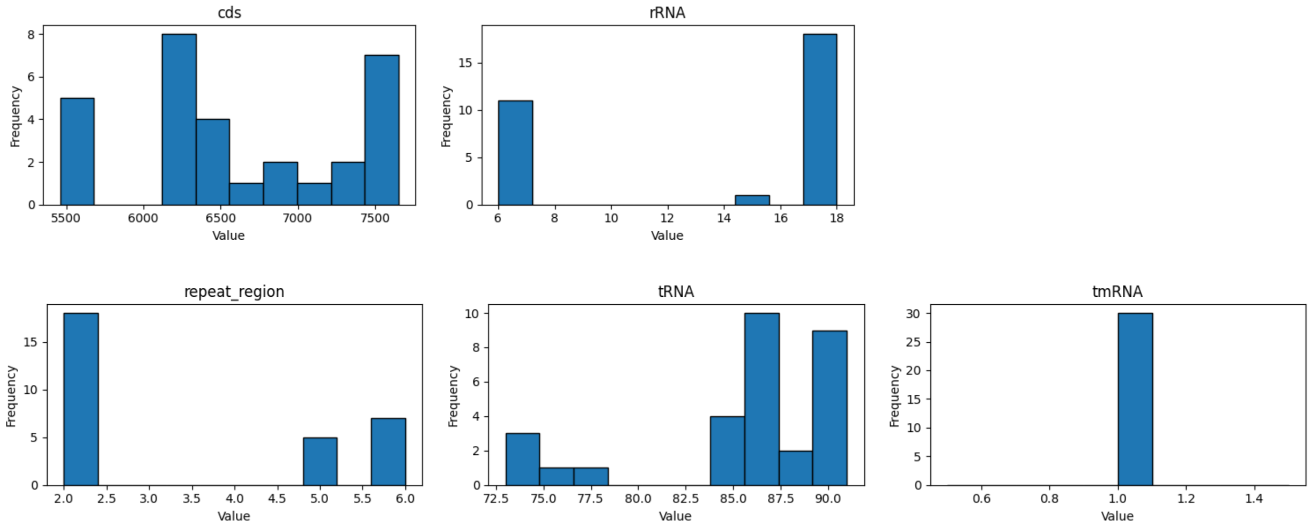

Figure S2. Histograms showing counting for each variable obtained from genome annotation of all 31 genomes. (CDS, rRNA, repeat regions, tRNA, and tmRNA).

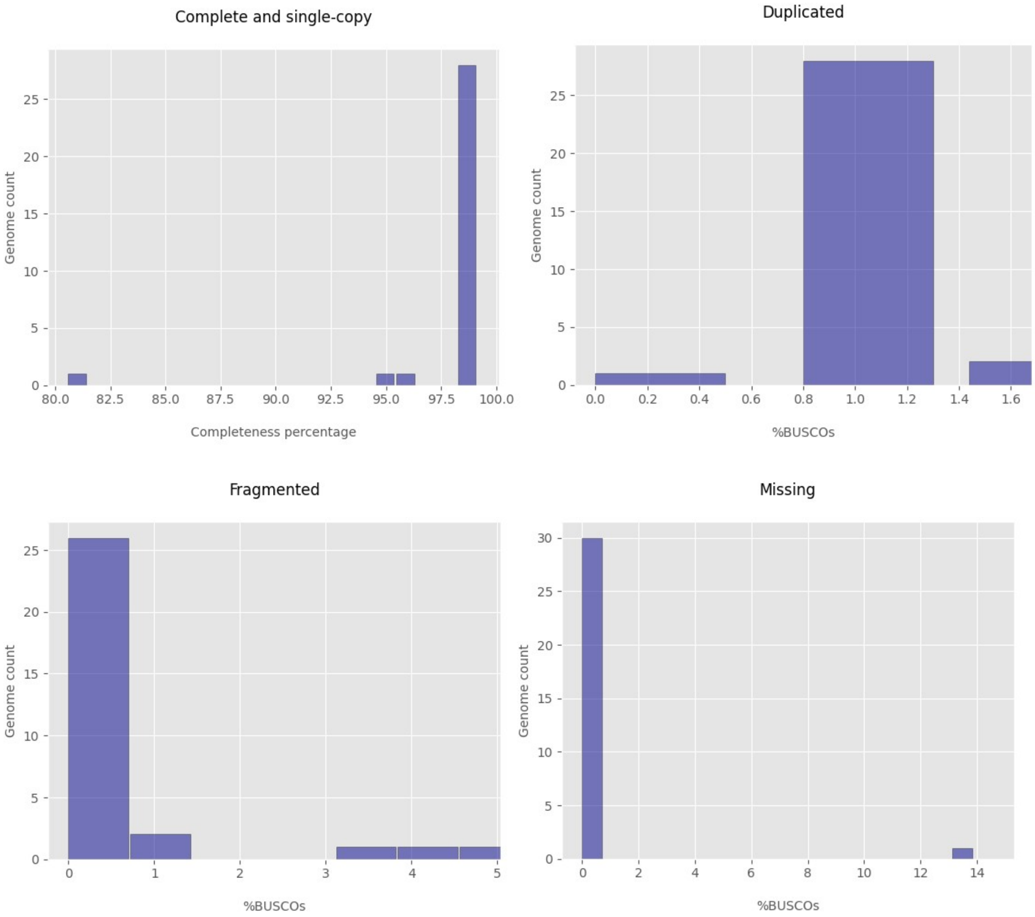

Figure S3. Completeness of all 31 genomes based on BUSCO analyses. One of the genomes considered does not fulfill the ≥ 95% threshold and hence not included in subsequent analyzes.

A)

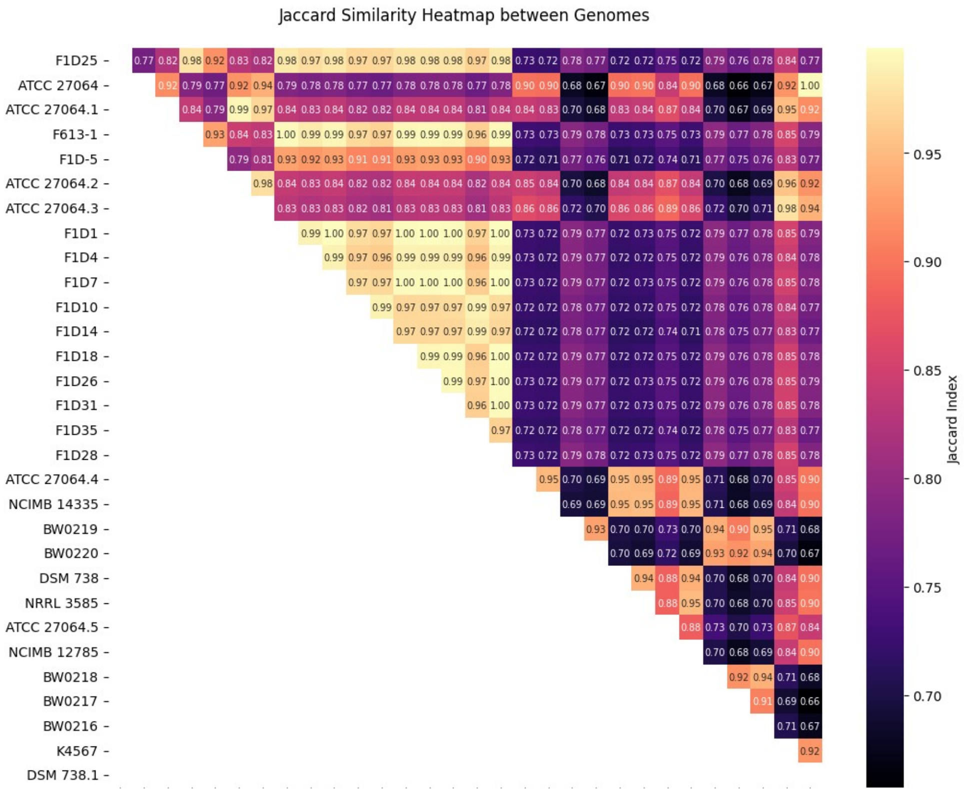
Jaccard Similarity Heatmap between Genomes
F1D25
ATCC 27064
ATCC 27064.1
F613-1
F1D-5
ATCC 27064.2
ATCC 27064.3
F1D1
F1D4
F1D7
F1D10
F1D14
F1D18
F1D26
F1D31
F1D35
F1D28
ATCC 27064.4
NCIMB 14335
BW0219
BW0220
DSM 738
NRRL 3585
ATCC 27064.5
NCIMB 12785
BW0218
BW0217
BW0216
K4567
DSM 738.1
0.95
0.90
0.85
0.80
0.75
0.70
Jaccard Index

B)

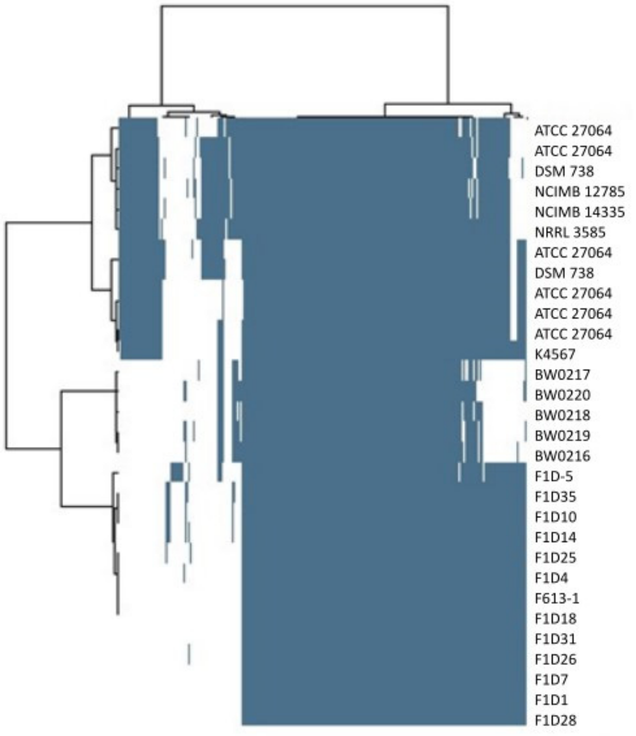
ATCC 27064
ATCC 27064
DSM 738
NCIMB 12785
NCIMB 14335
NRRL 3585
ATCC 27064
DSM 738
ATCC 27064
ATCC 27064
ATCC 27064
K4567
BW0217
BW0220
BW0218
BW0219
BW0216
F1D-5
F1D35
F1D10
F1D14
F1D25
F1D4
F613-1
F1D18
F1D31
F1D26
F1D7
F1D1
F1D28

C)

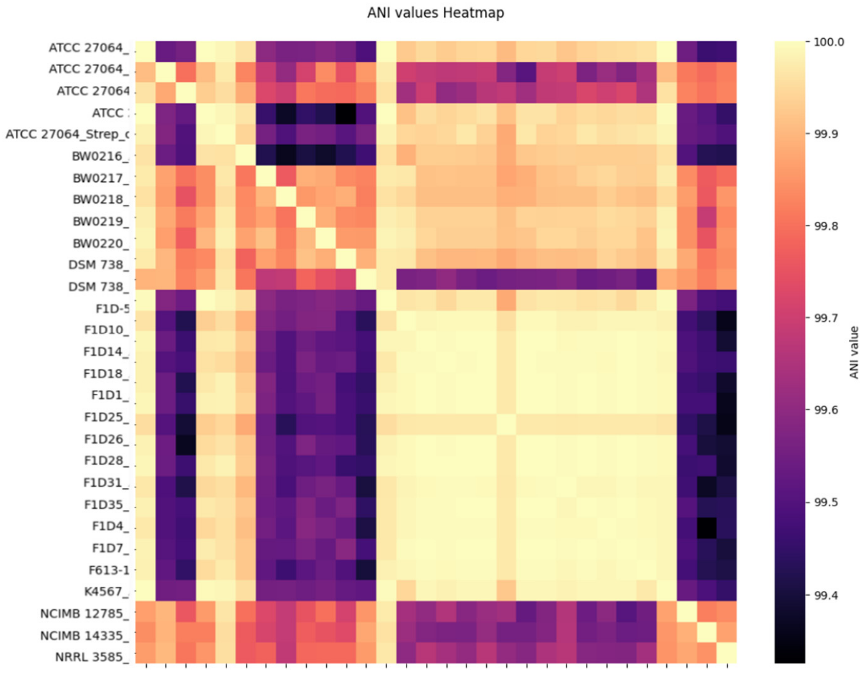

Figure S4. Gene content in terms of presence/absence of genes comparing the full set of 30 genomes in terms of the Jaccard index (A) - pairs with lower index are highlighted as black cells and indicate a lower number of shared genes, Manhattan distance (B) - A total of 8821 orthologous groups (genes) representing the full pangenome size are displayed, and Average Nucleotide Identity (ANI) (C) - ANI values oscillate between 99.3 and 100; the expected for strain-level comparison is ANI > %95. Gene presence is shown in blue and gene absence in white.

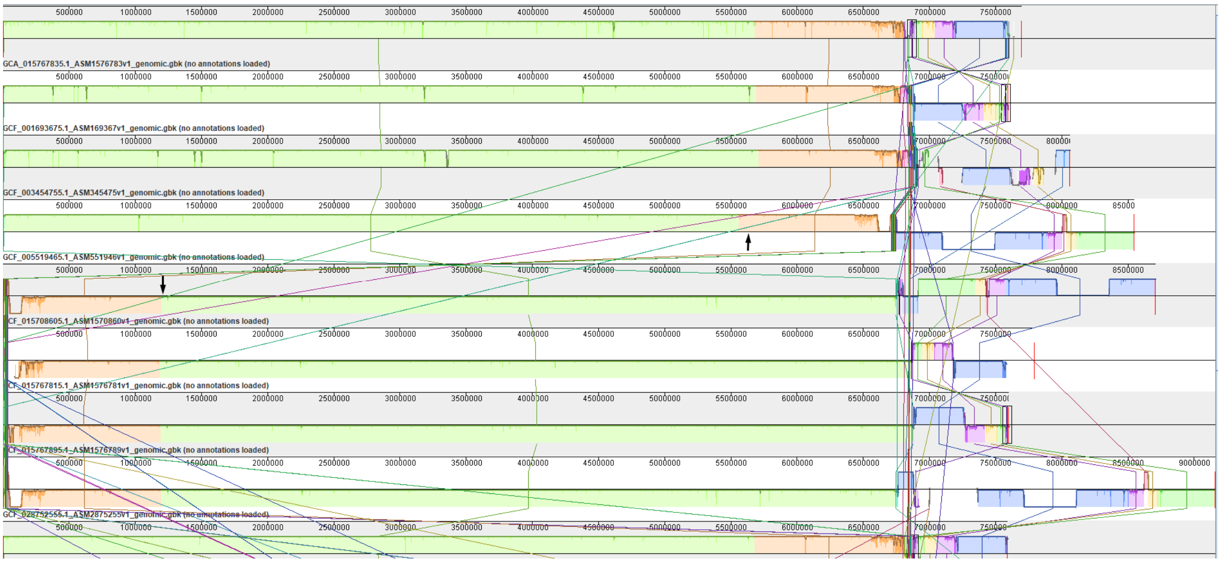

Figure S5. Synteny of 16 complete genomes of *S. clavuligerus* strains (only 9 are shown). Black arrows indicate an inversion of a region present in 4 genomes.

A)

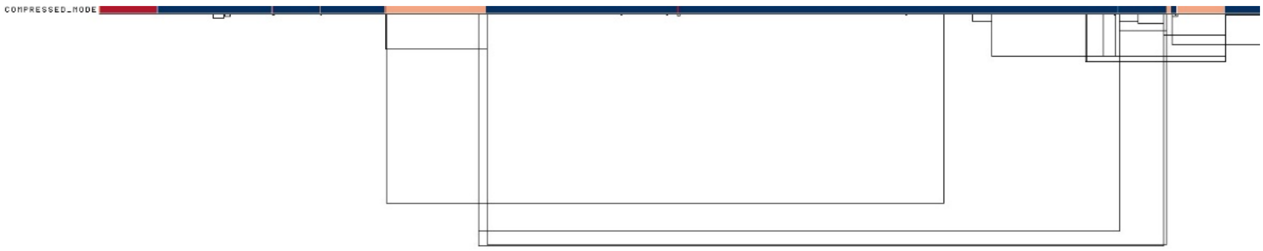
COMPRESSED_MODE

B)

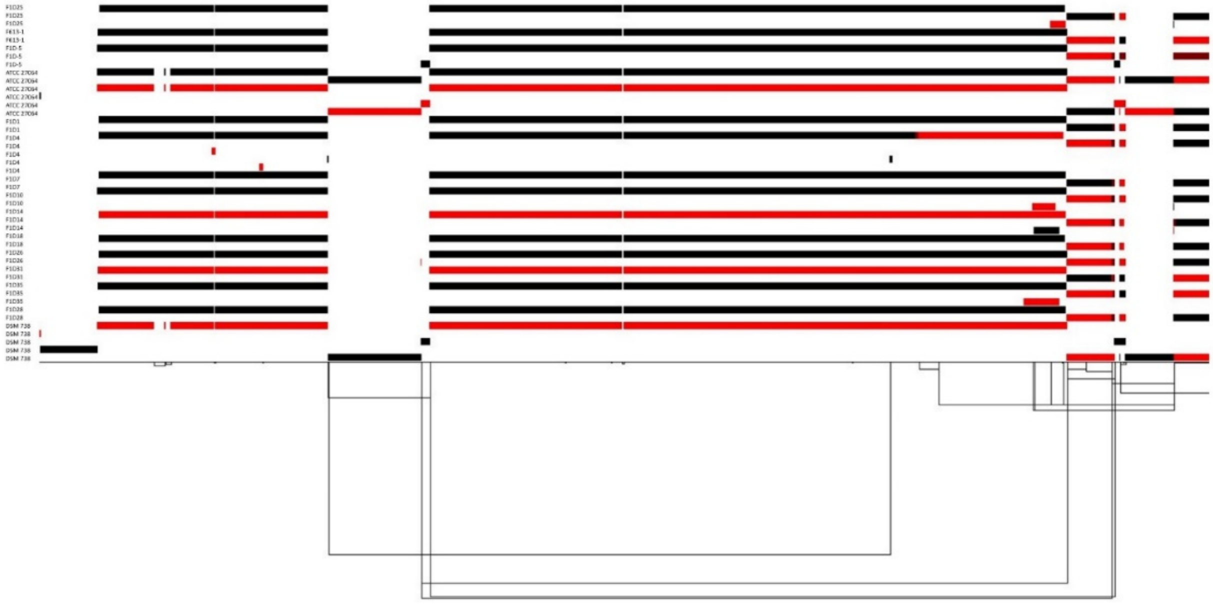
F1D25
F1D25
F1D25
FK13-1
FK13-1
F1D-5
F1D-5
F1D-5
ATCC 27064
ATCC 27064
ATCC 27064
ATCC 27064
ATCC 27064
ATCC 27064
F1D1
F1D1
F1D4
F1D4
F1D4
F1D4
F1D4
F1D7
F1D7
F1D10
F1D10
F1D14
F1D14
F1D14
F1D18
F1D18
F1D26
F1D26
F1D31
F1D31
F1D35
F1D35
F1D35
F1D28
F1D28
DSM 738
DSM 738
DSM 738
DSM 738
DSM 738

C)

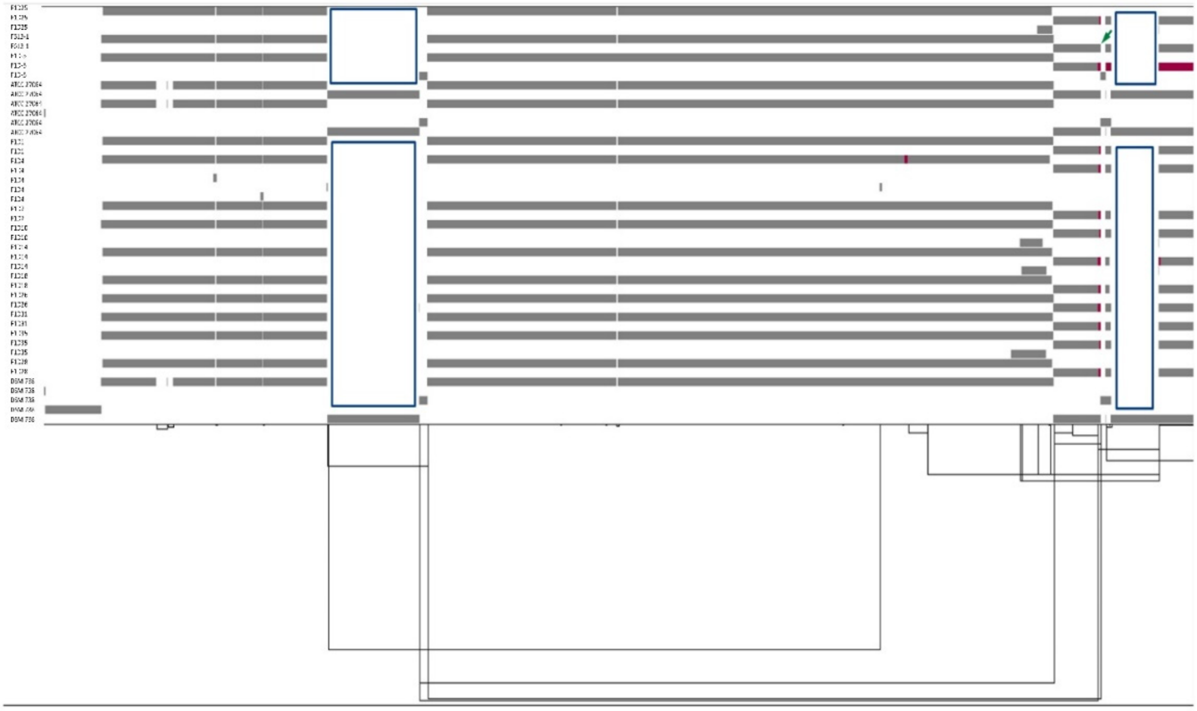

Figure S6. Graph visualization of the *S. clavuligerus* pan-genome. The conserved (blue) and non-conserved (red, rose) regions are displayed (**A**) where the lines correspond to the topology of the graph. (**B**) Graph visualization of the *S. clavuligerus* pan-genome comparing the different input genomes. The longer lines represent chromosomes while the shorter lines represent plasmid(s). Red coloring indicates a region of a given strain is differential compared to the group. Sequences of multiple genomes belonging to the same strain are displayed. The lines correspond to the topology of the graph. (**C**) The blue rectangles indicate plasmid regions that are unique in the group of input sequences. Missing plasmid regions are also shown (example case indicated by a green arrow). Differential regions are highlighted in red. The lines correspond to the topology of the graph.

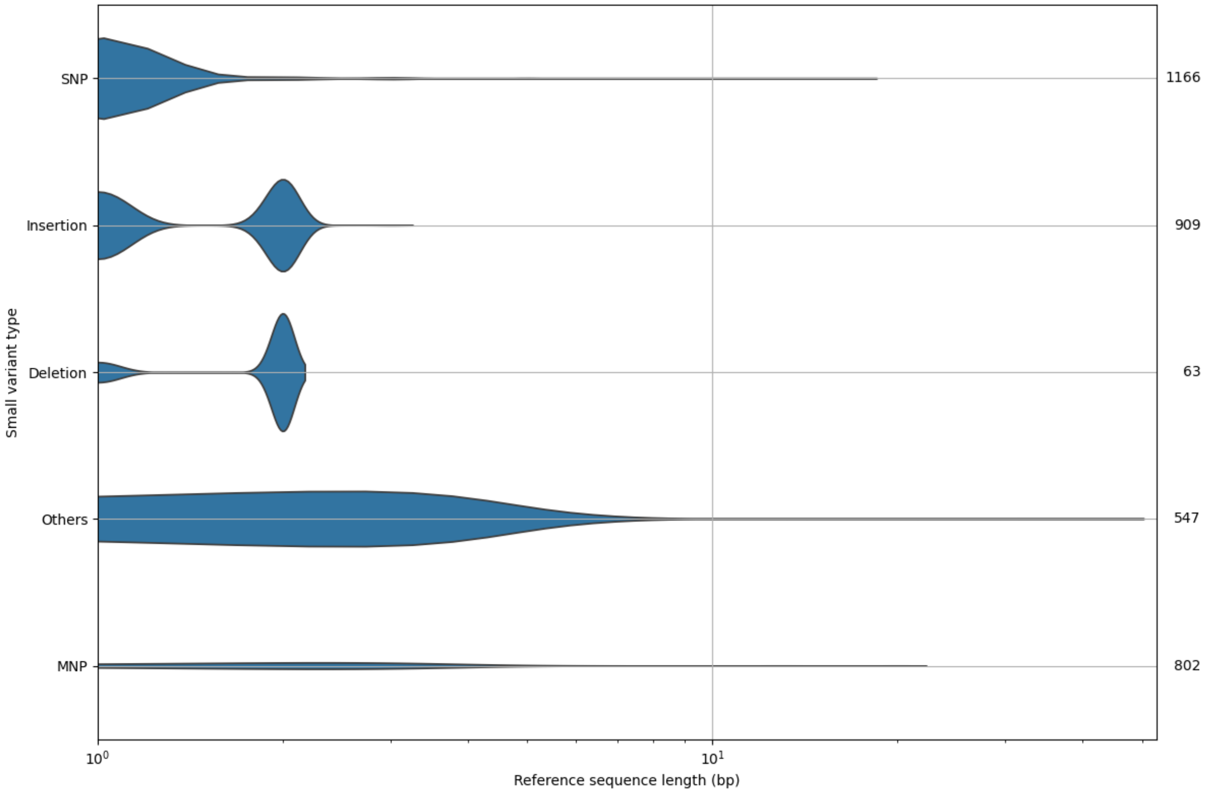

Figure S7. Classification of small (up to 10 bp long) nucleotide variants (Single Nucleotide Polymorphism, Insertion, Deletion, Multiple Nucleotide Polymorphism, Others) found in 30 *S. clavuligerus* genomes identified using one of the chromosome sequences as reference (Wild-type strain, GCF_015708605). The pan-genome graph representation output file was used as input while VG and annotation files were used for comparison among all genomes and mapping variants to coding regions within the reference genome.

A)

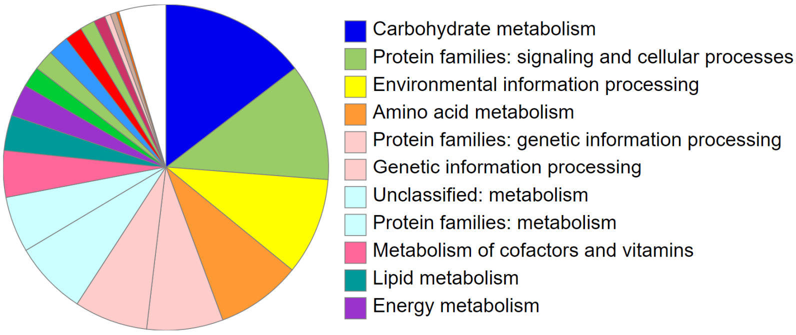

B)

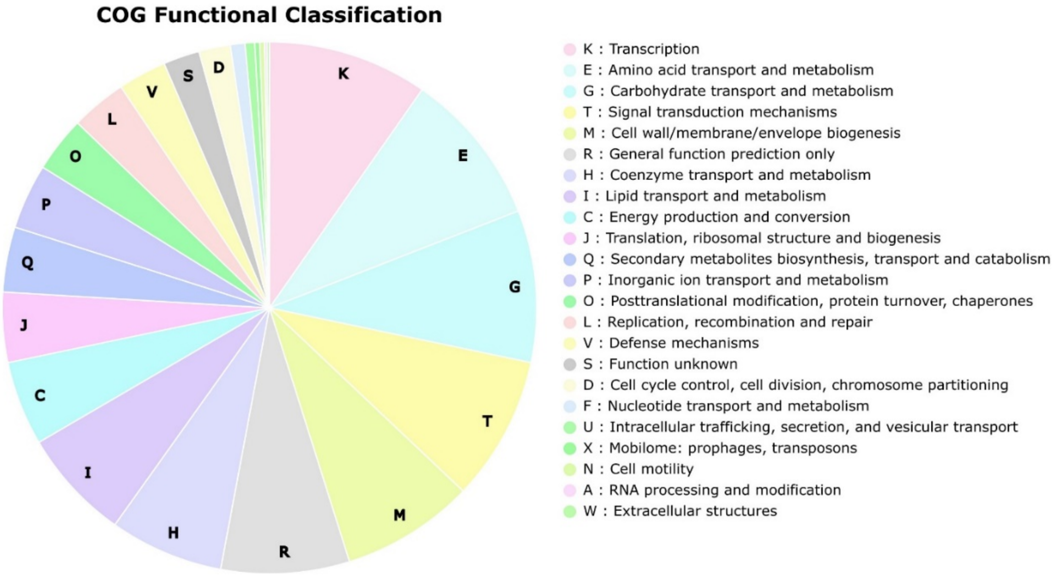

Figure S8. Functional annotation of *S. clavuligerus* variant containing genes. Functional annotation performed based on KEGG (**A**) and COG (**B**) databases of *S. clavuligerus* genes that contained nucleotide variants including Single Nucleotide Polymorphism (SNP), Insertion, Deletion, Multi Nucleotide Polymorphism (MNP), and cases in which multiple variation types occur.

A)

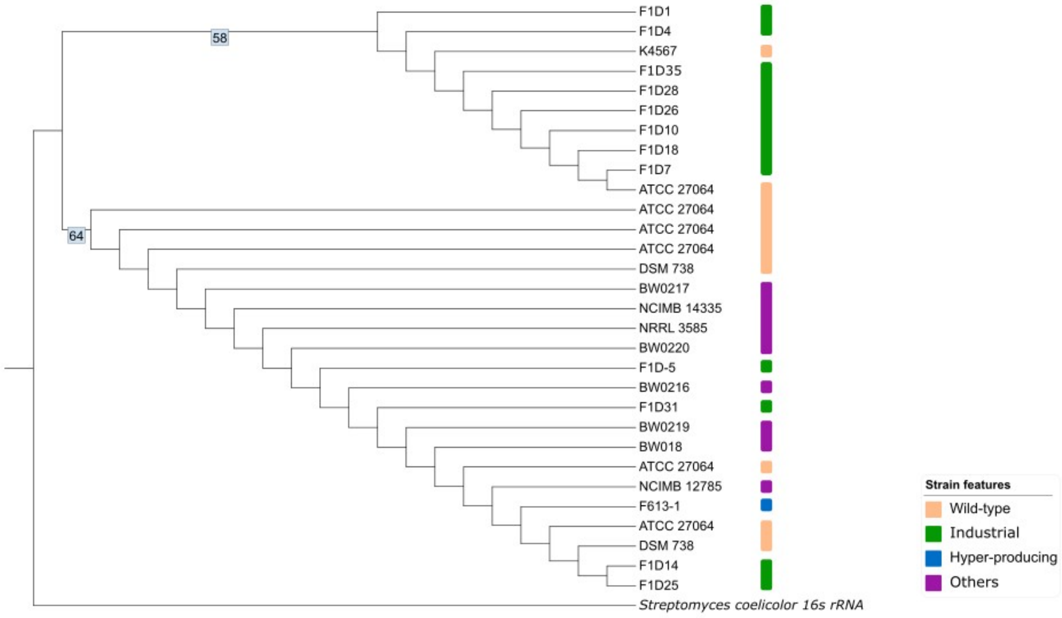

B)

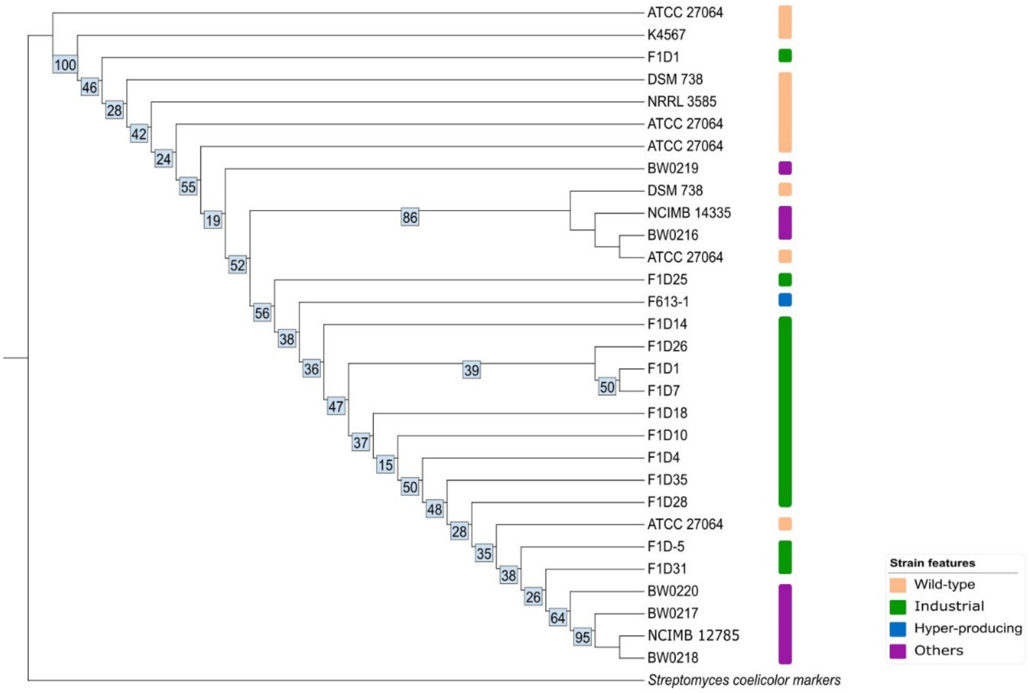

Figure S9. Evolutionary relationships of *S. clavuligerus* strains based on 16S rRNA (**A**) and concatenated 16S rRNA, *rpoB* and *gyrB* (**B**) sequences. Strain categories were defined as follows. Wild-type: Strains reported by the literature to be wild-type. Industrial: Strains known to be used in industrial CA production. Hyper-producing: Industrial strains for which CA produced has been published and significantly exceeds that produced by a wild-type strain. Others: Include strains known to have genetic modifications and/or whose relationship with wild-type strains is unclear and level of CA produced has not been published. Bootstrap values are used as branch support and highlighted in colored boxes. A missing branch support indicates a branch length of zero.

A)

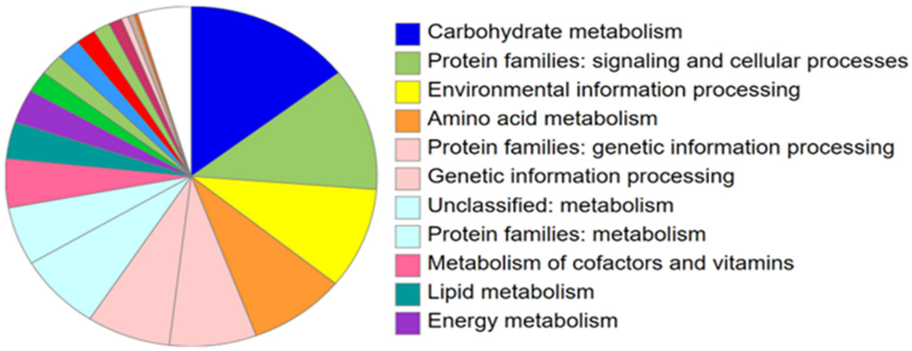

B)

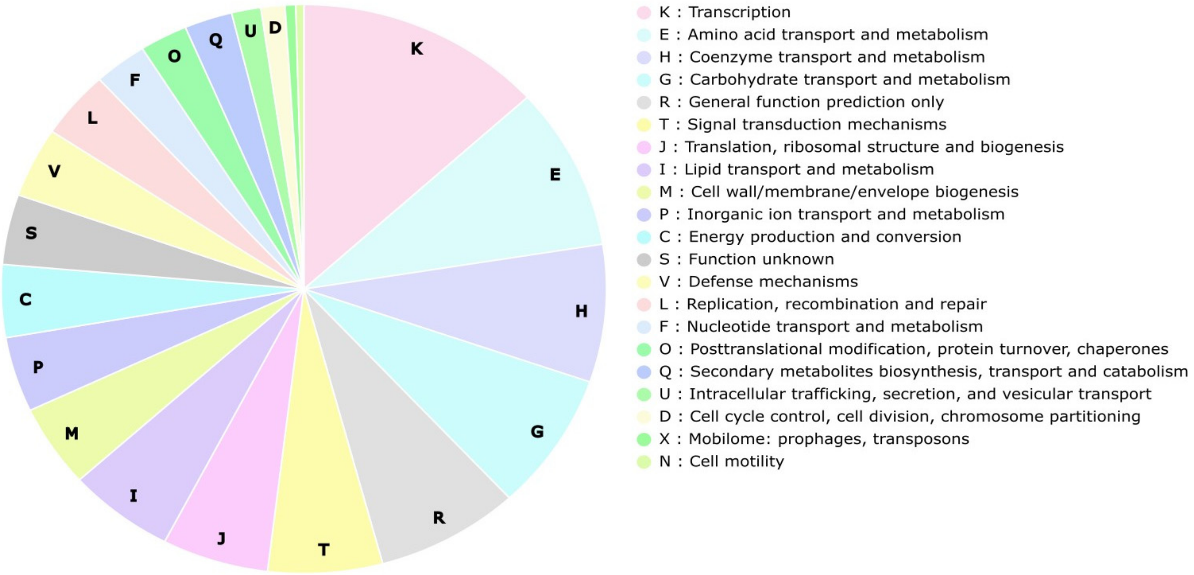

Figure S10. Functional annotation of *S. clavuligerus* fingerprint genes. Functional annotation based on KEGG (A) and COG (B) databases of potential fingerprint genes identified by comparison of 4,629 *S. clavuligerus* core genes against 145,452 Streptomycetes genes.

Figure S11. Presence/absence matrix per genome comparing the gene function annotation that antiSMASH related to genes in BGCs found in *S. clavuligerus* genomes (Please refer to SupplementaryFigureS11A.png file). The genome numbering corresponds to the same as Figure 11 while the gene function conventions can be found in SupplementaryFigureS11B.xlsx.

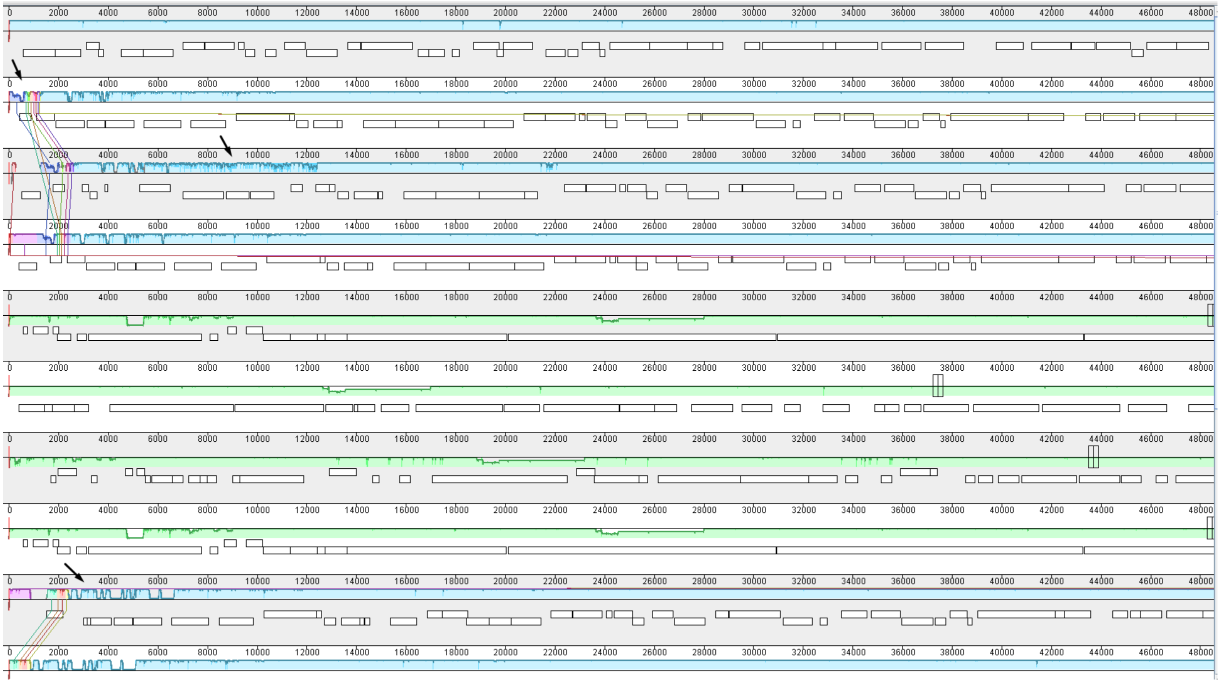

Figure S12. Synteny of 16 chromosomes obtained from complete genomes of *S. clavuligerus* strains (only 10 are shown). Black arrows indicate variation within small regions at one tip of the chromosomes.

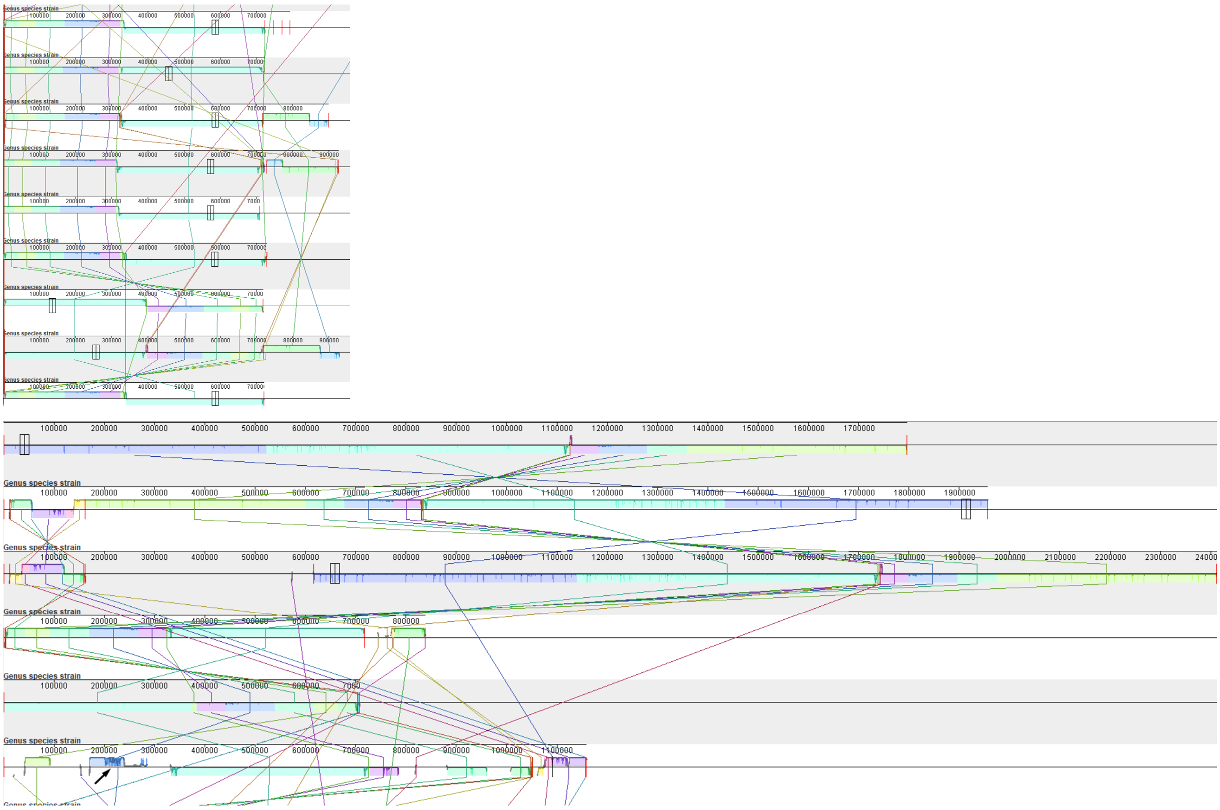

Figure S13. Synteny of plasmid content (some genomes included all pSCLn plasmids, while others included some of these or other plasmids) of 16 *S. clavuligerus* strain genomes. Black boxes indicate inversions while the back arrow indicates an example of a divergent region.

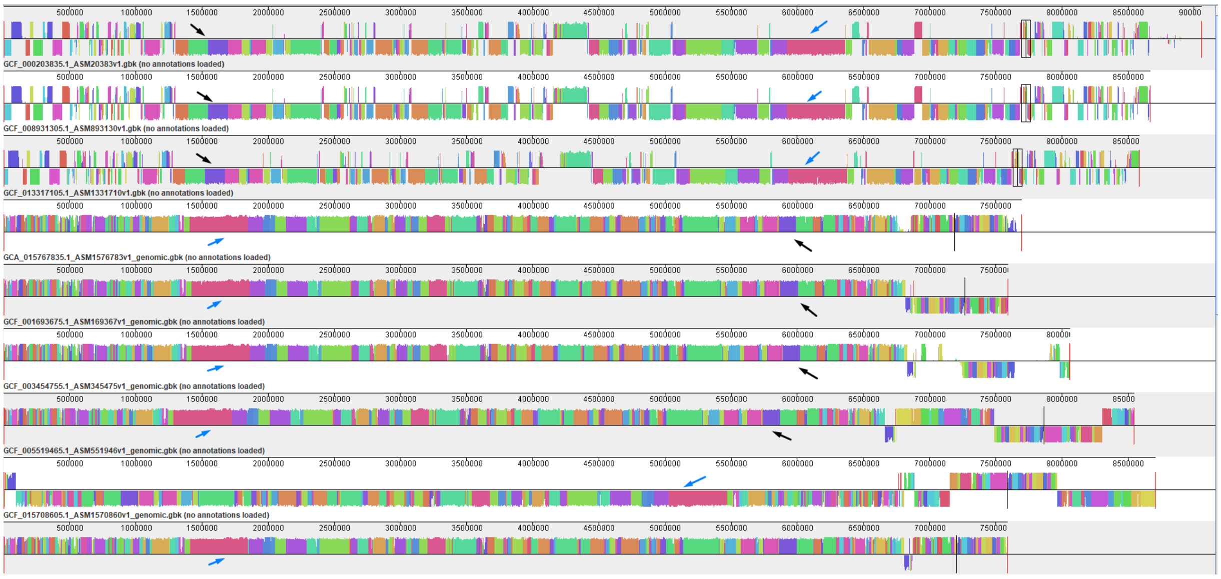

Figure S14. Synteny of 16 complete genomes of *S.clavuligerus* strains (only 6 are shown) and 3 complete genomes of *S. coelicolor* strains (at the top); black and blue arrows indicate examples of inversion of regions.

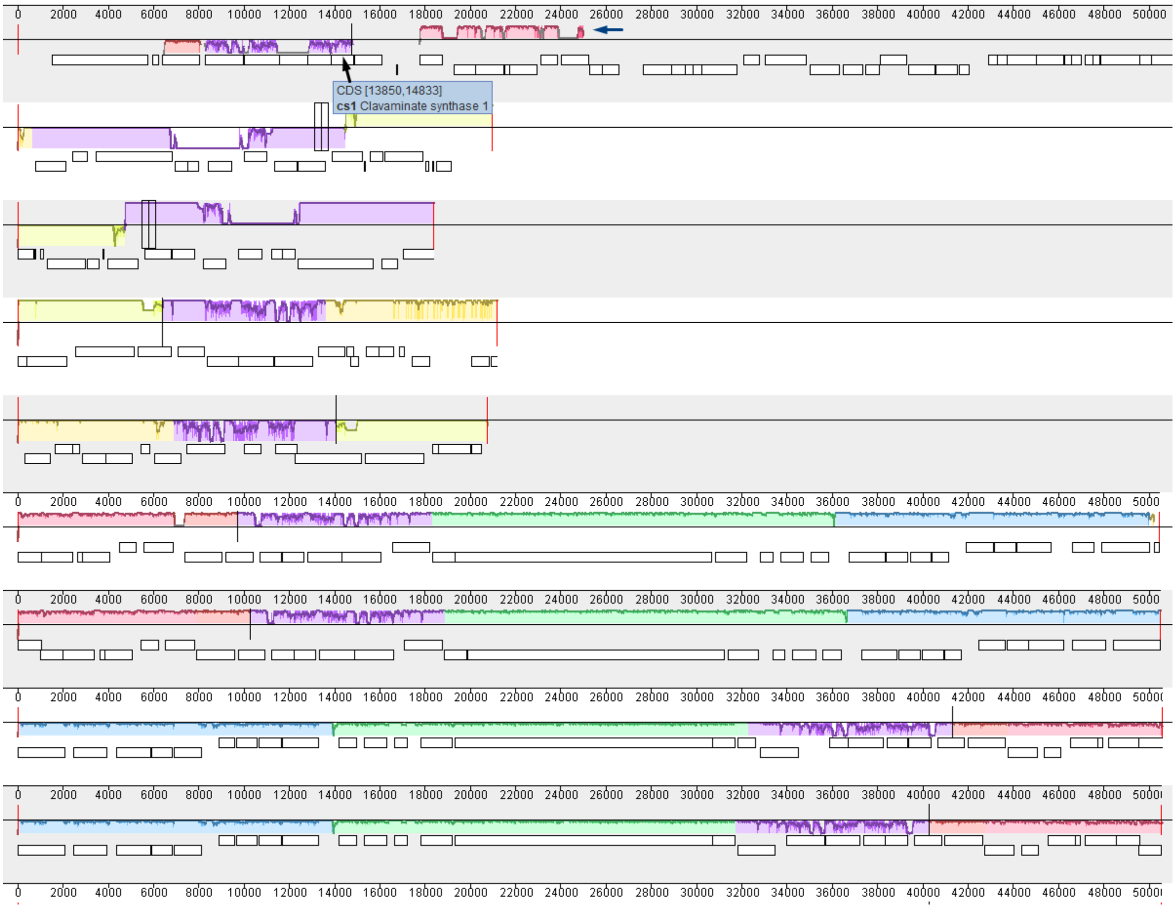

Figure S15. Two groups of sequences formed in synteny of regions associated with CA production (9 out of 78 regions are shown); short sequences of approximately 20kb and long sequences of approximately 45 kb. A black arrow indicates the position of Clavaminate synthase, a key enzyme in the biosynthesis of CA. A blue arrow indicates a region from *Saccharomonospora viridis* genome while the remaining regions correspond to *S. clavuligerus* genomes.

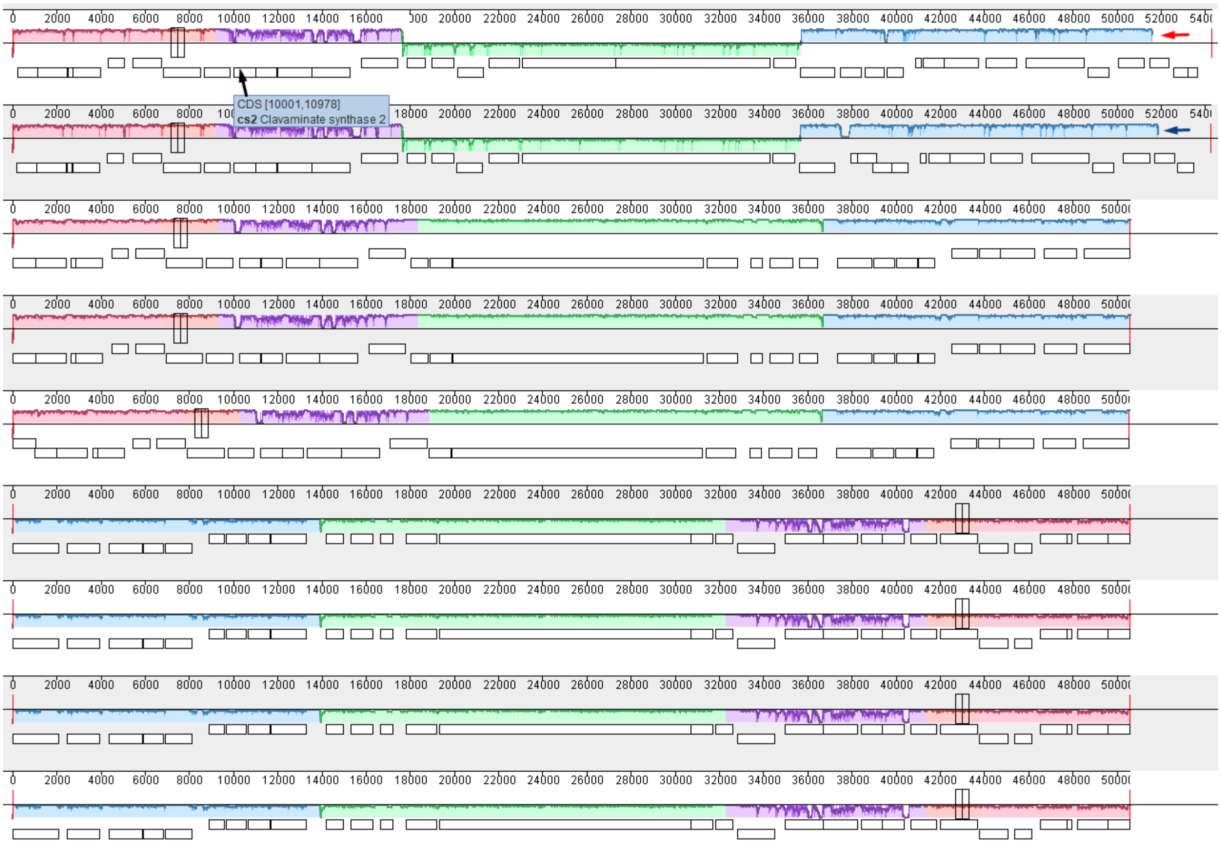

Figure S16. Similarity of regions associated with CA production (9 out of 78 regions are shown). A black arrow indicates the position of Clavaminate synthase, a key enzyme in the biosynthesis of CA. A red arrow indicates a region from *S. jumonjinensis* genome and a blue arrow indicates a region from *S. katsurahamanus* genome while the remaining regions correspond to *S. clavuligerus* genomes.

Table S1. Strain classification

Genome	Strain	Type	CA reported (mg L-1 at 120 H)	Source
GCF_001693675.1	F613-1	Hyperproducer	2250	https://doi.org/10.1186/s13568-019-0844-z
			525	https://doi.org/10.1128/spectrum.02017-22
GCF_019831915.1	K4567	Wild-type	556	https://doi.org/10.1007/s00253-022-11805-5
GCF_015912895.1	NRRL 3585	Wild-type	800	https://doi.org/10.1271/bbb.80569
GCF_000148465.1	ATCC 27064	Wild-type		
GCF_000163875.1	ATCC 27064	Wild-type		
GCF_003454755.1	F1D-5	Industrial		
GCF_005519465.1	ATCC 27064	Wild-type		
GCF_015708605.1	ATCC 27064	Wild-type		
GCF_015767735.1	F1D1	Industrial		
GCF_015767755.1	F1D4	Industrial		
GCF_015767775.1	F1D7	Industrial		
GCF_015767795.1	F1D10	Industrial		
GCF_015767815.1	F1D14	Industrial		
GCF_015767855.1	F1D18	Industrial		
GCF_015767875.1	F1D26	Industrial		

GCF_015767895.1	F1D31	Industrial
GCF_015767915.1	F1D35	Industrial
GCF_015767935.1	F1D28	Industrial
GCF_015912395.1	ATCC 27064	Wild-type
GCF_015912435.1	NCIMB 14335	Not wild-type, not modified
GCF_015912455.1	BW0219	Not industrial or hyperproduced but modified
GCF_015912465.1	BW0220	Not industrial or hyperproduced but modified
GCF_015912875.1	DSM 738	Wild-type
GCF_015912925.1	ATCC 27064	Wild-type
GCF_015912945.1	NCIMB 12785	Not wild-type, not modified
GCF_015912975.1	BW0218	Not industrial or hyperproduced but modified
GCF_015912985.1	BW0217	Not industrial or hyperproduced but modified
GCF_015912995.1	BW0216	Not industrial or hyperproduced but modified
GCA_028752555.1	DSM 738	Wild-type

* Wild-type, Hyperproducer, and Industrial categories indicate that strains have been reported as such. Remaining levels of column Type are considered as part of level Wild-type, in the manuscript; or Others, in Figure 3.

*'Genome' and 'CA reported' columns are independent. This means that values in 'CA reported' column corresponds to 'Strain' column yet may or may not correspond to 'Genome' column. 'Strain' and CA_produced columns are related, meaning that a given strain has been reported to produce a given amount of CA, this relation is supported by the 'Source' column. Empty rows indicate no reports of CA amount produced were found (at 120 H).

Table S2. Location and size of BGCs associated with β-lactam and/or CA biosynthesis in *S. clavuligerus* and other CA-producing species.

Species	Strain	Genome	Region	Size	Start	End
S. clavuligerus	ATCC 27064	GCF_000148465.1_Scla_1.0	NZ_CM001015.1.region010	18378	3428288	3446666
S. clavuligerus	ATCC 27064	GCF_000148465.1_Scla_1.0	NZ_CM001015.1.region012	50499	4838328	4888827
S. clavuligerus	ATCC 27064	GCF_000148465.1_Scla_1.0	NZ_CM001019.1.region010	21584	1144301	1165885
S. clavuligerus	ATCC 27064	GCF_000163875.1_Strep_clav_ATCC27064	NZ_CM000913.1.region010	18421	3436336	3454757
S. clavuligerus	ATCC 27064	GCF_000163875.1_Strep_clav_ATCC27064	NZ_CM000913.1.region012	50456	4846889	4897345
S. clavuligerus	ATCC 27064	GCF_000163875.1_Strep_clav_ATCC27064	NZ_CM000914.1.region010	21584	1143785	1165369
S. clavuligerus	ATCC 27064	GCF_005519465.1_ASM551946v1	NZ_CP027858.1.region015	50543	1861959	1912502
S. clavuligerus	ATCC 27064	GCF_005519465.1_ASM551946v1	NZ_CP027858.1.region017	20975	3301353	3322328
S. clavuligerus	ATCC 27064	GCF_005519465.1_ASM551946v1	NZ_CP027859.1.region010	21584	1142864	1164448

S. clavuligerus	ATCC 27064	GCF_015708605.1_ASM1570860v1	NZ_CP045847.1.region010	18385	3428215	3446600
S. clavuligerus	ATCC 27064	GCF_015708605.1_ASM1570860v1	NZ_CP045847.1.region012	50543	4836226	4886769
S. clavuligerus	ATCC 27064	GCF_015708605.1_ASM1570860v1	NZ_CP045850.1.region009	21584	630261	651845
S. clavuligerus	ATCC 27064	GCF_015912395.1_ASM1591239v1	NZ_WKJU01000008.1.region001	36722	0	36722
S. clavuligerus	ATCC 27064	GCF_015912395.1_ASM1591239v1	NZ_WKJU01000117.1.region001	15045	0	15045
S. clavuligerus	ATCC 27064	GCF_015912925.1_ASM1591292v1	NZ_WKJT01000007.1.region001	36741	590	37331
S. clavuligerus	ATCC 27064	GCF_015912925.1_ASM1591292v1	NZ_WKJT01000096.1.region001	15148	0	15148
S. clavuligerus	BW0216	GCF_015912995.1_ASM1591299v1	NZ_WMCC01000013.1.region001	28735	0	28735
S. clavuligerus	BW0217	GCF_015912985.1_ASM1591298v1	NZ_WMCB01000005.1.region001	36721	0	36721
S. clavuligerus	BW0218	GCF_015912975.1_ASM1591297v1	NZ_WMCA01000008.1.region001	36722	0	36722
S. clavuligerus	BW0219	GCF_015912455.1_ASM1591245v1	NZ_WMBZ01000006.1.region001	36722	0	36722
S. clavuligerus	BW0220	GCF_015912465.1_ASM1591246v1	NZ_WMBY01000005.1.region001	36715	0	36715
Saccharomonospora viridis	DSM 43017	GCF_000023865.1	NC_013159.1.region006	81229	3621257	3702486
S. clavuligerus	DSM 738	GCF_015912875.1_ASM1591287v1	NZ_WKJW01000016.1.region001	28729	0	28729
S. clavuligerus	DSM 738	GCF_015912875.1_ASM1591287v1	NZ_WKJW01000226.1.region001	10403	0	10403
S. clavuligerus	DSM 738	GCF_028752555.1_ASM2875255v1	NZ_CP086229.1.region010	18385	3428209	3446594
S. clavuligerus	DSM 738	GCF_028752555.1_ASM2875255v1	NZ_CP086229.1.region012	50543	4836220	4886763
S. clavuligerus	DSM 738	GCF_028752555.1_ASM2875255v1	NZ_CP086233.1.region010	21584	1142938	1164522
S. clavuligerus	F1D1	GCF_015767735.1_ASM1576773v1	NZ_CP065246.1.region016	50589	1986866	2037455
S. clavuligerus	F1D1	GCF_015767735.1_ASM1576773v1	NZ_CP065246.1.region018	20975	3427313	3448288
S. clavuligerus	F1D1	GCF_015767735.1_ASM1576773v1	NZ_CP065247.1.region005	21584	283117	304701
S. clavuligerus	F1D14	GCF_015767815.1_ASM1576781v1	NZ_CP065258.1.region010	18385	3416275	3434660
S. clavuligerus	F1D14	GCF_015767815.1_ASM1576781v1	NZ_CP065258.1.region012	50589	4825292	4875881
S. clavuligerus	F1D14	GCF_015767815.1_ASM1576781v1	NZ_CP065259.1.region005	21584	271767	293351
S. clavuligerus	F1D18	GCF_015767855.1_ASM1576785v1	NZ_CP065234.1.region016	50589	1986936	2037525
S. clavuligerus	F1D18	GCF_015767855.1_ASM1576785v1	NZ_CP065234.1.region018	20975	3427383	3448358
S. clavuligerus	F1D18	GCF_015767855.1_ASM1576785v1	NZ_CP065235.1.region005	21584	271422	293006
S. clavuligerus	F1D25	GCA_015767835.1_ASM1576783v1	CP065236.1.region016	50601	1983610	2034211
S. clavuligerus	F1D25	GCA_015767835.1_ASM1576783v1	CP065236.1.region018	21005	3423821	3444826
S. clavuligerus	F1D25	GCA_015767835.1_ASM1576783v1	CP065237.1.region005	21584	284088	305672
S. clavuligerus	F1D26	GCF_015767875.1_ASM1576787v1	NZ_CP065239.1.region016	50589	1991954	2042543
S. clavuligerus	F1D26	GCF_015767875.1_ASM1576787v1	NZ_CP065239.1.region018	20975	3432399	3453374
S. clavuligerus	F1D26	GCF_015767875.1_ASM1576787v1	NZ_CP065240.1.region005	21584	283459	305043
S. clavuligerus	F1D28	GCF_015767935.1_ASM1576793v1	NZ_CP065244.1.region016	50589	1986869	2037458
S. clavuligerus	F1D28	GCF_015767935.1_ASM1576793v1	NZ_CP065244.1.region018	20975	3427316	3448291
S. clavuligerus	F1D28	GCF_015767935.1_ASM1576793v1	NZ_CP065245.1.region005	21584	283821	305405

S. clavuligerus	F1D31	GCF_015767795.1_ASM1576779v1	NZ_CP065255.1.region016	50589	1998794	2049383
S. clavuligerus	F1D31	GCF_015767795.1_ASM1576779v1	NZ_CP065255.1.region018	20975	3439240	3460215
S. clavuligerus	F1D31	GCF_015767795.1_ASM1576779v1	NZ_CP065256.1.region005	21584	283980	305564
S. clavuligerus	F1D31	GCF_015767895.1_ASM1576789v1	NZ_CP065261.1.region010	18385	3422565	3440950
S. clavuligerus	F1D31	GCF_015767895.1_ASM1576789v1	NZ_CP065261.1.region012	50589	4831582	4882171
S. clavuligerus	F1D31	GCF_015767895.1_ASM1576789v1	NZ_CP065262.1.region004	21584	416990	438574
S. clavuligerus	F1D35	GCF_015767915.1_ASM1576791v1	NZ_CP065241.1.region015	50589	1997243	2047832
S. clavuligerus	F1D35	GCF_015767915.1_ASM1576791v1	NZ_CP065241.1.region017	20975	3437689	3458664
S. clavuligerus	F1D35	GCF_015767915.1_ASM1576791v1	NZ_CP065242.1.region004	21584	413373	434957
S. clavuligerus	F1D4	GCF_015767755.1_ASM1576775v1	NZ_CP065248.1.region016	50589	1990777	2041366
S. clavuligerus	F1D4	GCF_015767755.1_ASM1576775v1	NZ_CP065248.1.region018	20975	3430660	3451635
S. clavuligerus	F1D4	GCF_015767755.1_ASM1576775v1	NZ_CP065249.1.region005	21584	283950	305534
S. clavuligerus	F1D-5	GCF_003454755.1_ASM345475v1	NZ_CP032052.1.region016	50364	1999420	2049784
S. clavuligerus	F1D-5	GCF_003454755.1_ASM345475v1	NZ_CP032052.1.region018	20975	3454305	3475280
S. clavuligerus	F1D-5	GCF_003454755.1_ASM345475v1	NZ_CP032054.1.region004	21195	285580	306775
S. clavuligerus	F1D-5	GCF_003454755.1_ASM345475v1	NZ_CP032054.1.region008	20747	745432	766179
S. clavuligerus	F1D7	GCF_015767775.1_ASM1576777v1	NZ_CP065253.1.region016	50589	1988326	2038915
S. clavuligerus	F1D7	GCF_015767775.1_ASM1576777v1	NZ_CP065253.1.region018	20975	3428773	3449748
S. clavuligerus	F1D7	GCF_015767775.1_ASM1576777v1	NZ_CP065254.1.region005	21584	279372	300956
S. clavuligerus	F613-1	GCF_001693675.1_ASM169367v1	NZ_CP016559.1.region016	50543	1997701	2048244
S. clavuligerus	F613-1	GCF_001693675.1_ASM169367v1	NZ_CP016559.1.region018	20975	3437137	3458112
S. clavuligerus	F613-1	GCF_001693675.1_ASM169367v1	NZ_CP016560.1.region004	21584	401850	423434
S. clavuligerus	K4567	GCF_019831915.1_ASM1983191v1	NZ_JADANO010000002.1.region016	50543	1998499	2049042
S. clavuligerus	K4567	GCF_019831915.1_ASM1983191v1	NZ_JADANO010000002.1.region018	20975	3437893	3458868
S. clavuligerus	K4567	GCF_019831915.1_ASM1983191v1	NZ_JADANO010000003.1.region009	21584	627842	649426
S. clavuligerus	NCIMB 12785	GCF_015912945.1_ASM1591294v1	NZ_WKJX01000011.1.region001	28731	0	28731
S. clavuligerus	NCIMB 12785	GCF_015912945.1_ASM1591294v1	NZ_WKJX01000099.1.region001	15098	0	15098
S. clavuligerus	NCIMB 14335	GCF_015912435.1_ASM1591243v1	NZ_WKJY01000007.1.region001	36719	0	36719
S. clavuligerus	NCIMB 14335	GCF_015912435.1_ASM1591243v1	NZ_WKJY01000085.1.region001	15014	0	15014
S. clavuligerus	NRRL 3585	GCF_015912895.1_ASM1591289v1	NZ_WKJV01000011.1.region001	28729	0	28729
S. clavuligerus	NRRL 3585	GCF_015912895.1_ASM1591289v1	NZ_WKJV01000199.1.region001	10420	0	10420
Streptomyces jumonjinensis	NRRL 5741	GCF_009600885.1	NZ_VCLA01000156.1.region003	54281	106552	160833
Streptomyces katsurahamanus	T-272	GCF_009600895.1	NZ_VDEQ01000103.1.region001	54259	9873	64132

* Start and End columns represent the exact location of regions that contain BGCs within a given genome.

Table S3. Sequences and classification of CDSs and PFAM domains identified within the CA cluster in *S. clavuligerus* genomes (Please refer to SupplementaryTableS14.xlsx file).
